# *Polygonatum sibiricum* Delar. ex Redoute: A Potential Functional Food for the Prevention and Treatment of Hyperuricemia

**DOI:** 10.1002/fsn3.70985

**Published:** 2025-10-11

**Authors:** Peng Zhao, Wenjing Liu, Minyang He, Weiyou Cao, Qichang Xu, Qing Hao, Lijun Wang, Ying Liu, Jingyu Wang, Feixue Wang, Lin Jiang, Songze Li, Chunyan Dai, Qiusheng Zheng, Jun Ma, Xiangcheng Fan, Jichun Han

**Affiliations:** ^1^ College of Traditional Chinese Medicine Binzhou Medical University Yantai China; ^2^ Department of Cardiac Surgery I Yantai Yuhuangding Hospital Yantai China; ^3^ Department of Gastroenterology, the Fourth Affiliated Hospital of School of Medicine, and International School of Medicine, International Institutes of Medicine Zhejiang University Yiwu China; ^4^ Department of Pathology, The Fourth Affiliated Hospital of School of Medicine, and International School of Medicine, International Institutes of Medicine Zhejiang University Yiwu China; ^5^ Department of Pharmacy, Center for X Medicine, the Fourth Affiliated Hospital of School of Medicine, and International School of Medicine, International Institutes of Medicine Zhejiang University Yiwu China; ^6^ Center for Innovative Traditional Chinese Medicine Target and New Drug Research International Institutes of Medicine, Zhejiang University Yiwu China

**Keywords:** food‐medicine homology, functional food, hyperuricaemia, *Polygonatum sibiricum* Delar. ex Redoute, uric acid

## Abstract

This study evaluated the antihyperuricemic potential of *Polygonatum sibiricum* (HJ) in both animal and human models. A mouse hyperuricemia (HUA) model was established by administering a high uric acid (UA) diet. The model was then treated with HJ water extract. Serum levels of UA, creatinine (Cr), and urea nitrogen (BUN) were measured using biochemical assay kits. Kidney tissue damage was assessed through H&E and PAS staining. Network pharmacology analysis was employed to elucidate potential mechanisms of action. Untargeted metabolomics analysis was performed on mouse serum using liquid chromatography‐mass spectrometry. HJ water extract significantly reduced serum UA levels in HUA mice from 700.82 μmol/L to 236.61 μmol/L (66% decrease). The treatment also markedly lowered serum creatinine and BUN levels, inhibited xanthine oxidase activity, and alleviated renal and glomerular histopathology. Network pharmacology identified MAOA and CYP3A4 among key targets, implicating regulation of histidine metabolism and steroid hormone biosynthesis. Untargeted metabolomics further revealed HJ‐induced alterations in tyrosine metabolism, amino acid biosynthesis, and histidine pathways. Western blotting confirmed downregulation of MAOA and CYP1A1 proteins. These findings suggest dual mechanisms of action: MAOA‐mediated modulation of histidine metabolism and CYP enzyme‐mediated adjustment of steroid hormone synthesis. In a pilot clinical study, 30 HUA volunteers consuming 0.2 g HJ daily for 8 weeks showed a progressive reduction in serum UA by approximately 22% without adverse effects. These results support the development of HJ as a functional food ingredient for the prevention and treatment of HUA. Further research is warranted to fully elucidate the mechanisms of action and optimize dosing regimens for clinical application.

## Graphical abstract

This study demonstrates that *Polygonatum sibiricum* (HJ) exhibits significant therapeutic potential for managing hyperuricemia (HUA), primarily through mechanisms involving the regulation of histidine metabolism via MAOA targets and the modulation of steroid hormone biosynthesis via CYP3A4, CYP1A2, and CYP1A1 targets.

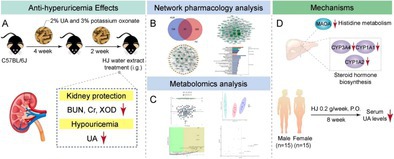

**Abbreviations**
BUN: urea nitrogen
Cr: creatinine
GO: gene ontology analysis
HJ: *Polygonatum sibiricum* Delar. ex Redoute
HJ‐H: 10 mg/kg HJ water extract group
HJ‐L: 0.1 mg/kg HJ water extract group
HJ‐M: 1 mg/kg HJ water extract group
HUA: hyperuricemia
KEGG: kyoto encyclopedia of genes and genomes
OMIM: on‐line mendelian inheritance in man
OPLS‐DA: orthogonal partial least squares discriminant analysis
TCMIP: integrative pharmacology‐based research platform of traditional Chinese medicine
TTD: therapeutic target database
UA: uric acid
VIP: variable importance in projection
XOD: xanthine oxidase

## 1 Introduction

Hyperuricemia (HUA) is a common metabolic disease caused by disorders in purine metabolism. Under normal dietary conditions, if fasting blood uric acid (UA) levels measured on non‐consecutive days exceed 420 μmol/L in men and 360 μmol/L in women, it is diagnosed as HUA (Zhang et al. 2021). Currently, HUA levels have been closely linked to the occurrence and development of several diseases, including gout, kidney stones, cardiovascular diseases, type 2 diabetes, and metabolic syndrome. Notably, HUA is the primary risk factor for gout, with approximately 12% of HUA patients progressing to this painful arthritic condition (Du et al. 2024). The prevalence of HUA in China has reached alarming levels. According to the “2021 China Hyperuricemia and Gout Trend White Paper,” the overall prevalence of HUA in China is 13.3%, affecting approximately 177 million people. Of these, about 8.3% progress to gout. Hyperuricemia has emerged as the second most prevalent metabolic disorder following diabetes mellitus, with an increasing incidence observed in younger populations (Cheng‐Yuan and Jian‐Gang 2023). Therefore, there is an urgent need to develop safe and effective prevention and treatment methods for HUA.

Currently, medications used clinically to treat HUA can be categorized into three main types: UA synthesis inhibitors, UA excretion promoters, and UA decomposition promoters (Liu et al. 2024). While these pharmacological interventions can be effective, they are often associated with significant side effects and limitations, particularly with long‐term use. UA synthesis inhibitors, such as xanthine oxidase (XOD) inhibitors, reduce the conversion of hypoxanthine and xanthine to UA by inhibiting the activity of XOD (Zhao et al. 2022). Although these medications can lower UA levels, long‐term use may lead to reductions in white blood cells and platelets. UA excretion promoters, like allopurinol, may be poorly tolerated by some patients; meanwhile, benzbromarone can effectively inhibit renal tubular reabsorption of UA, accelerating its excretion. However, long‐term use of these drugs can increase the risk of liver toxicity. Additionally, benzbromarone can cause gastrointestinal side effects such as abdominal cramping, fever, and headache. UA decomposition promoters, such as uricase, are enzymes that promote the oxidative degradation of UA into allantoin, reducing tubular reabsorption and facilitating its excretion. While uricase is commonly used in clinical practice, it has limitations, including high costs and potential allergic reactions (Piani et al. 2023; Sun et al. 2024). Given these challenges, there is a pressing need for therapeutic agents that are not only effective in managing HUA but also have minimal side effects and are cost‐effective.

The concept of food‐medicine homology, rooted in traditional Chinese medicine, offers a promising avenue for HUA management. This approach, articulated in the ancient text “Huangdi Neijing,” posits that certain substances can serve as both food and medicine, depending on the context of their use. Food‐medicine homologues are natural resources that have been identified through centuries of human experience as capable of both satisfying nutritional needs and regulating bodily functions to prevent or treat diseases (Chen 2023; Huang et al. 2023).

Modern scientific research has corroborated the therapeutic potential of various food‐medicine homologues in managing HUA. For instance, chicory has been shown to lower UA levels by inhibiting xanthine oxidase and adenine deaminase, two key enzymes in purine metabolism (Chen 2023; Ji et al. 2022). Celery extract can reduce UA production through XOD inhibition (Dolati et al. 2018), while chrysanthemum extract rich in luteolin has demonstrated significant UA‐lowering effects in clinical studies (Cheng‐Yuan and Jian‐Gang 2023; Ng et al. 2022). Other foods with potential anti‐HUA properties include lotus leaves, mint, yam, Radix Notoginseng, rice shoots, Poria, and *Terminalia chebula* . Compared to conventional pharmaceuticals, these food‐medicine homologues often exhibit lower toxicity, better long‐term tolerability, and a more holistic approach to disease management, making them promising candidates for HUA treatment and prevention.

*Polygonatum sibiricum* Delar. ex Redoute, a perennial herb of the Asparagaceae family, is known as “huangjing” in China (referred to as HJ in this study). It has a long history of use in traditional Chinese medicine and is recognized as a food‐medicine homologue with diverse therapeutic properties. HJ is traditionally used to replenish qi and nourish yin, strengthen the spleen, moisten the lungs, and benefit the kidneys (Xu et al. 2023). It is commonly prescribed for conditions that share symptomatic overlap with HUA, including spleen qi deficiency, fatigue, dry mouth, reduced appetite, and various manifestations of internal heat and deficiency (Chen et al. 2021).

Despite the apparent correlation between the traditional uses of HJ and the clinical manifestations of HUA, research specifically investigating its potential in preventing and treating HUA is lacking. Therefore, this study aims to evaluate the antihyperuricemic effects of HJ using both animal models and a pilot human study. By combining in vivo experiments with network pharmacology and metabolomics analyses, we seek to not only demonstrate the efficacy of HJ in managing HUA but also to elucidate its potential mechanisms of action. The findings of this study could provide valuable evidence supporting the development of HJ as a functional food ingredient for HUA management, offering a safe, effective, and potentially more tolerable alternative to conventional pharmacological interventions.

## 2 Materials and Methods

### 2.1 Chemicals and Materials

HJ was purchased from Huayu Agricultural Development Co. Ltd. (Yantai, Shandong Province, China). The authenticity of the plant material was verified by Associate Professor Jingping Yu, an expert in traditional Chinese medicine resources at Binzhou Medical University. The UA Content Assay Kit (catalog number: bc1365), Urea Nitrogen (BUN) Content Assay Kit (catalog number: bc1535), Creatinine (Cr) Content Assay Kit (catalog number: bc4910), and Xanthine Oxidase (XOD) Activity Assay Kit (catalog number: bc1095) were purchased from Beijing Solarbio Science and Technology Co. Ltd. (Beijing, China). Potassium sulfonate (CAS: 2207‐75‐2, purity ≥ 99.0%) and hypoxanthine (CAS: 68‐94‐0, purity ≥ 99.0%) were purchased from Beijing Solarbio Science and Technology Co. Ltd. (Beijing, China). Untargeted metabolomics detection‐related reagents were obtained from the Shenzhen Weike Meng Technology Group Co. Ltd. (Shenzhen, China). All other chemicals and reagents used were of analytical grade.

### 2.2 Preparation of HJ Extract

**FIGURE 1:**
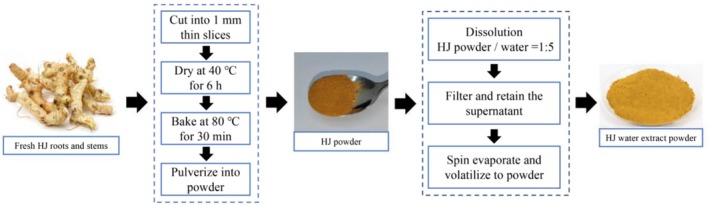
Flow chart of HJ water extract preparation. The preparation process of *Polygonatum sibiricum* (HJ) water extract: Fresh HJ roots and stems were sliced into 1 mm thin pieces, dried at 40°C for 6 h, and baked at 80°C for 30 min until golden yellow. The dried material was then ground into powder. The powder was dissolved in water at a 1:5 ratio, filtered to retain the supernatant, and the liquid was evaporated using a rotary evaporator to obtain the HJ water extract powder.

The HJ extract was prepared following a standardized process (Figure 1): (1) Fresh HJ roots and stems were thoroughly cleaned and air‐dried. (2) The dried HJ slices were baked at 80°C until they turned golden yellow, yielding roasted HJ rhizome slices. (3) 100 g of roasted HJ rhizomes were ground into a fine powder. (4) The HJ powder was boiled in purified water (1:5 ratio), and the resulting filtrate was collected. The liquid extract was then concentrated using an RE2000E rotary evaporator (Shanghai Yarong Biochemistry Instrument Factory, China). This process yielded 13.72 g of HJ water extract powder, representing an extraction rate of 13.72% (w/w).

### 2.3 Animals and Ethics Statement

A total of 50 adult male C57BL/6 mice (4 weeks old) were used in this study. All animals were purchased from Sibeifu (Beijing) Biotechnology Co. Ltd. License number: SCXK (jing) 2019–0010. Animals were housed in steel rodent cages under controlled environmental conditions: temperature maintained at 22°C ± 2°C, humidity at 60%–80%, and a 12‐h light/dark cycle (light period: 07:00–19:00; dark period: 19:00–07:00). The animal use protocol listed below was reviewed and approved by the Binzhou Medical University Institutional Animal Care and Use Committee (2023‐378). The study was conducted in strict accordance with institutional guidelines for the care and use of laboratory animals.

### 2.4 Mouse HUA Model

This study used a special diet to construct a mice HUA model. Special feed contains 2% (w/w) UA and 3% (w/w) potassium oxonate, provided by Jiangsu Xiehe Pharmaceutical Bioengineering Co. Ltd. After feeding C57BL/6 mice with special feed for 4 weeks, UA levels increased and kidney tissue was damaged, establishing a mice HUA model.

### 2.5 Design of the Animal Experimental Groups

**FIGURE 2:**
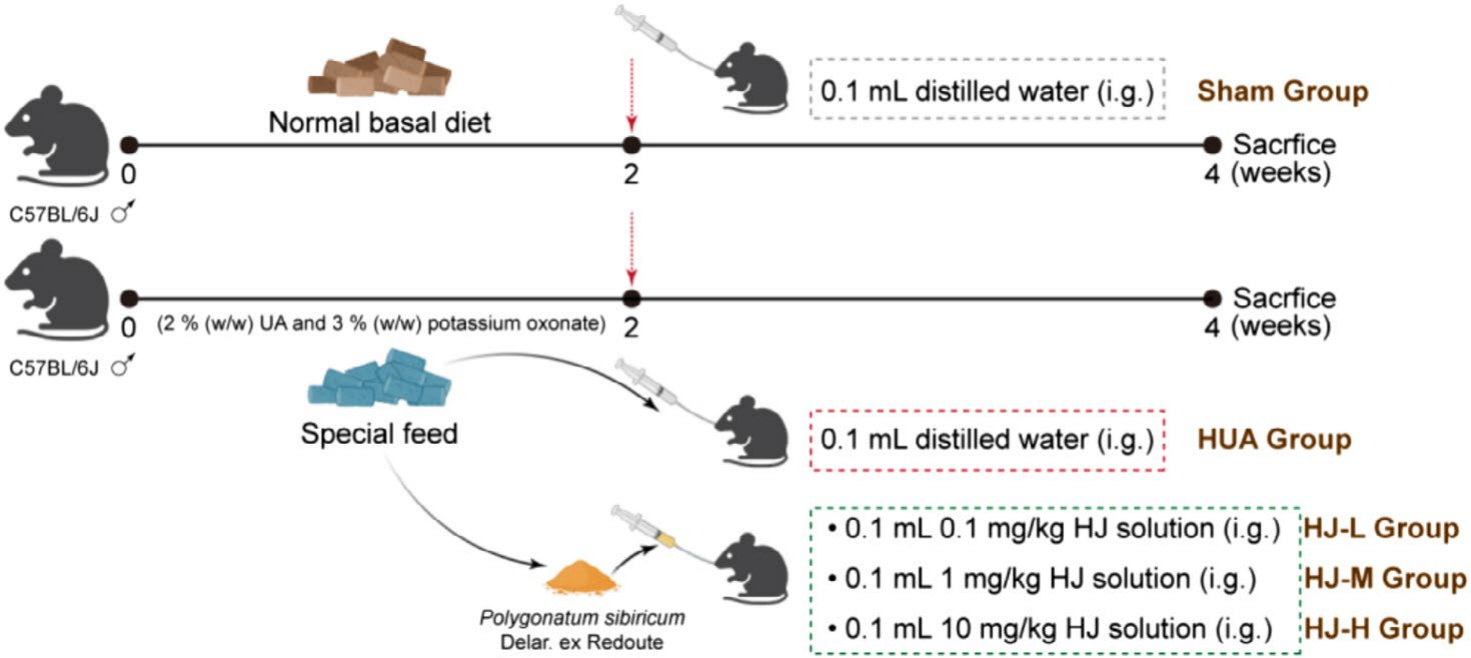
Grouping processing flowchart. The experimental design used to evaluate the effects of various treatments on C57BL/6J male mice. Mice are divided into two main groups: the Sham Group, which receives a normal basal diet along with 0.1 mL distilled water (i.g.) for 4 weeks, and the HUA Group, which receives a special feed containing 2% (w/w) uric acid (UA) and 3% (w/w) potassium oxonate, followed by different treatments with varying doses of HJ solution (0.1 mL at 0.1 mg/kg, 1 mg/kg, and 10 mg/kg, i.g.). All mice are sacrificed after 4 weeks of treatment.

Fifty adult male C57BL/6 mice were randomly divided into 5 groups: sham group, HUA group, 0.1 mg/kg HJ water extract group (HJ‐L), 1 mg/kg HJ water extract group (HJ‐M), and 10 mg/kg HJ water extract group (HJ‐H). The treatment for each experimental group was as follows (Figure 2): (1) In the sham group, after feeding C57BL/6 mice with a normal basal diet for 2 weeks, start gavage of 0.1 mL distilled water once a day and continue feeding them with a normal basal diet for 2 weeks. (2) In the HUA group, after feeding C57BL/6 mice with special feed for 2 weeks, start gavage of 0.1 mL distilled water once a day and continue feeding them with special feed for 2 weeks. (3) In the HJ‐L group, after feeding C57BL/6 mice with special feed for 2 weeks, start daily gavage of 0.1 mg/kg HJ water extract (dissolve the required dose of HJ water extract in 0.1 mL distilled water), and continue feeding special feed for 2 weeks. (4) In the HJ‐M group, after feeding C57BL/6 mice with special feed for 2 weeks, start daily gavage of 1 mg/kg HJ water extract (dissolve the required dose of HJ water extract in 0.1 mL distilled water), and continue feeding special feed for 2 weeks. (5) In the HJ‐H group, after feeding C57BL/6 mice with special feed for 2 weeks, start daily gavage of 10 mg/kg HJ water extract (dissolve the required dose of HJ water extract in 0.1 mL distilled water), and continue feeding special feed for 2 weeks.

### 2.6 Biochemical Index Detection

After the model was established, 0.5 mL of blood was taken from the mice eyeball, left at room temperature for 2 h, centrifuged (4000 *g*) for 10 min, collected, packaged, and stored at −20°C for future use. By strictly following the instructions of the UA, Cr, BUN, and XOD test kits, the UA, Cr, BUN, and XOD levels in the serum were determined.

### 2.7 Detecting Pathological Damage to the Kidneys and Glomeruli

After blood collection, the C57BL/6 mice were euthanized via the cervical dislocation method, and their livers were removed. The livers were washed with precooled physiological saline and dried with filter paper. The livers were then fixed with 4% (w/v) neutral formaldehyde. H&E‐stained sections were used to observe pathological damage in renal tissue, whereas periodic acid‐Schiff (PAS)‐stained sections were used to observe pathological damage in glomeruli.

### 2.8 Prediction of Potential Compounds and Related Targets of HJ

Through the Integrative Pharmacology‐based Research Platform of Traditional Chinese Medicine (TCMIP v2.0, http://www.tcmip.cn/TCMIP/index.php) based on ligand characteristics, protein structure, and relevant publications, data mining was conducted to identify the active ingredients of HJ (Xu et al. 2019). A large number of literature reviews were conducted to supplement the confirmed active ingredients of compounds that were not included in the TCMIP database and to predict the potential targets of HJ.

### 2.9 Screening of Potential Targets for HUA

In GeneCards database (https://www.genecards.org/), OMIM database (https://www.omim.org/), DrugBank database (https://go.drugbank.com/), and TTD database (http://db.idrblab.net/ttd/), screen genes related to HUA. Enter the key phrase “hyperuricemia” in each database to obtain disease‐related targets, and delete duplicate data to obtain the final gene list.

### 2.10 Network Establishment and Protein–Protein Interaction (PPI) Analysis

The compound target network (C‐T network) was constructed using Cytoscape v3.9.0 software (Bai et al. 2024). Import the specific data of key active compounds and their corresponding targets of HJ into Cytoscape v3.9.0 software, and use the network analysis tool in the software to calculate the network topology. Key evaluation parameters of the network can be obtained, such as “betweenness centrality”, “closeness centrality”, “degree centrality”, etc. Select the key active compounds of HJ for analysis.

Venn was created using online tools from bioinformatics and evolutionary genomics (http://bioinformatics.psb.ugent.be/webtools/Venn/). Import HJ related goals and HUA related goals separately, and obtain common goals through Venn analysis. Using Venn to compare and analyze the targets corresponding to HJ and those related to HUA, the common targets obtained by the intersection of the two are considered as potential therapeutic targets for HJ treatment of HUA. Using STRING v11.5 (https://cn.string‐db.org/), commonly used targets were used for PPI analysis (Gu et al. 2024). The species is restricted to “Homo sapiens”, with a minimum required interaction score of > 0.4. Clear the disconnected nodes in the PPI network, and then download the TSV file of the network. Import TSV files into Cytoscape v3.9.0 software for visual analysis, and screen and identify corresponding target proteins using degree as a parameter. Each node corresponds to a gene or an effective compound of HJ, connected by a straight line. The more effective compounds a gene corresponds to, the larger the nodes of the gene. The nodes of compounds derived from different traditional Chinese medicines are represented by different colors, while compounds from multiple sources or shared targets are represented by corresponding mixed colors. Finally, the core gene targets were obtained through topological analysis.

### 2.11 KEGG and GO Analysis

Use Metscape (https://metascape.org/gp/index.html) software for KEGG analysis (Zhou et al. 2019). Set the following information, such as a minimum enrichment value of 1.5, a minimum overlap value of 3, and a *p*‐value cutoff value of 0.01. Finally, the results will be exported for future research and visualized using an online tool called SRplot (http://www.Bioinformatics.com.cn/srplot) for data analysis and visualization.

Perform GO enrichment analysis using DAVID v6.8 (https://david.ncifcrf.gov/home.jsp) (Sherman et al. 2022). There are three types of GO enrichment: biological processes (BP), cellular components (CC), and molecular functions (MF), which describe the different functions of genes and their products, respectively.

### 2.12 Untargeted Serum Metabolomics Detection

Untargeted metabolomics analysis was performed on the serum of each group of mice using liquid chromatography–mass spectrometry. The analysis was conducted using an Agilent 1290 Infinity LC system (Agilent Technologies, USA) coupled with a 6460 Triple Quadrupole Mass Spectrometer (Agilent Technologies, USA). The serum was processed and analyzed according to the standard procedures for metabolomics. After the model was established, 1 mL of blood was collected from the mouse eyeball, left at room temperature for 2 h, and centrifuged at 4000 g for 10 min, after which 150 μL of serum was collected. The serum was then sent to the Weike Meng Technology Company (Tianjing, China) for untargeted metabolomics analysis.

### 2.13 Case Collection

Patient source: 30 patients with HUA, who were admitted to the Affiliated Hospital of Binzhou Medical College from March 2023 to October 2023, including 15 males and 15 females aged between 30 and 65 years, were selected as the research group. The medical ethics committee of Binzhou Medical University approved the study. (2025‐001).

Patient selection criteria: These criteria were developed on the basis of the American College of Rheumatology (ACR) gout classification criteria of 1977 and the ACR/EULAR gout classification criteria of 2015.

The inclusion criteria for hyperuricaemia were as follows: (1) met the diagnostic criteria for hyperuricaemia, which means that the diagnostic criterion for hyperuricaemia was that the fasting blood UA levels were measured twice on different days, with levels > 420 μmol/L in males and levels > 360 μmol/L in females; (2) ages between 40 and 65 years, the sex of which is not limited; (3) acute onset, with a course of disease within 14 days; and (4) no gout symptoms have yet appeared. (5) All participants provided informed consent and signed an informed consent form.

The exclusion criteria were as follows: not taking diuretics or medications to lower UA levels within 3 months; acute or chronic infections; hypertension; coronary heart disease; malignant tumors; liver and kidney diseases; diabetes; blood system diseases; abnormal liver and kidney function or gout; and pregnancy or lactation.

The case dropout criteria were as follows: (1) patients who experienced serious adverse events or complications and were not suitable for continued treatment; (2) patients who had poor compliance; who used medication that did not reach 80% of the prescribed amount or exceeded 120% of the prescribed amount; (3) patients who voluntarily withdrew or were lost to follow‐up during the treatment process; (4) patients who had not completed the entire course of treatment, which affected the efficacy or safety assessment; and (5) patients whose incomplete information affected the validity and safety of the judgment.

Patient dropout treatment: (1) After the subject falls off, the researcher should try to contact the subject as much as possible, inquire about the reasons, and complete the evaluation items as much as possible. (2) The dropout cases are all required for the set of statistics to provide a full analysis, and dropout patients do not need to be supplemented separately.

The termination test criteria were as follows: (1) Those who experienced serious adverse events; (2) during the course of the disease, the condition worsened, and ineffective cases were treated; (3) serious deviations occurred during the implementation of clinical trial protocols; and (4) the subjects requested to withdraw during the clinical trial process.

### 2.14 HJ Efficacy Testing

A total of 30 HUA volunteers, including 15 males and 15 females, were selected for this study. Volunteers drank HJ each day for 8 weeks, with a daily intake of 0.2 g. Weekly testing of serum UA levels in HUA volunteers was performed.

### 2.15 Safety Evaluation Criteria

According to the “Guidelines for Clinical Research of Traditional Chinese Medicine New Drugs (Trial)” published in 2002, the main focus of interest is the digestive disorders and abdominal pain of patients before and after treatment, as well as other adverse reactions, such as gastrointestinal discomfort, bloating, vomiting, diarrhea, bloody stools, and black stools, and safety indicators, such as liver and kidney function. The degrees of symptoms were classified as follows: (1) no adverse reactions; (2) mild reactions were mild, short‐lived, and tolerable; (3) moderate reactions were severe, stopped, and could be relieved without treatment; and (4) severe reactions, terminated, were symptomatic.

### 2.16 Statistical Analysis

The data are presented as the means ± standard deviations from at least six independent experiments. Statistical differences were determined by using Student's *t*‐tests, where *p* < 0.05 was considered to indicate statistical significance. The analyses were performed via the Statistical Program for Social Sciences Software (International Business Machines Corporation, New York, USA).

## 3 Results

### 3.1 HJ Reduces HUA in Mice

To evaluate the anti‐HUA effects of HJ, we assessed serum levels of UA, blood BUN, Cr, and XOD activity. As shown in Table 1, the HUA group exhibited significantly higher serum levels of UA, Cr, and BUN, along with elevated XOD activity, compared to the sham group. In contrast, treatment with HJ at both medium (HJ‐M) and high (HJ‐H) doses resulted in markedly reduced serum levels of UA, Cr, and BUN, as well as decreased XOD activity in HUA mice, compared to the untreated HUA group.

**TABLE 1:** HJ significantly reduced the levels of UA (A), Cr (B), BUN (C), and XOD activity (D) in the serum of HUA mice (*n* = 10).

Group	UA (μmol/L)	Cr (μmol/L)	BUN (μmol/L)	XOD (U/L)
Sham	248.39 + 34.47	69.82 + 8.49	4.27 + 0.63	7.03 + 1.23
HUA	700.82 + 68.22^a^	167.90 + 16.18^a^	9.90 + 1.69^a^	16.19 + 1.64^a^
HJ‐L	685.55 + 58.42	148.86 + 11.44	7.44 + 0.50	15.33 + 1.79
HJ‐M	377.27 + 53.36^b^	104.38 + 12.41^b^	5.21 + 0.53^b^	12.71 + 1.15^b^
HJ‐H	236.61 + 58.09^b^	81.00 + 7.18^b^	4.08 + 0.52^b^	10.68 + 1.00^b^

^a^ *p* < 0.01 compared to the sham group.

^b^ *p* < 0.01 compared to the HUA group.

### 3.2 HJ Alleviates Renal Tissue Damage in HUA Mice

**FIGURE 3:**
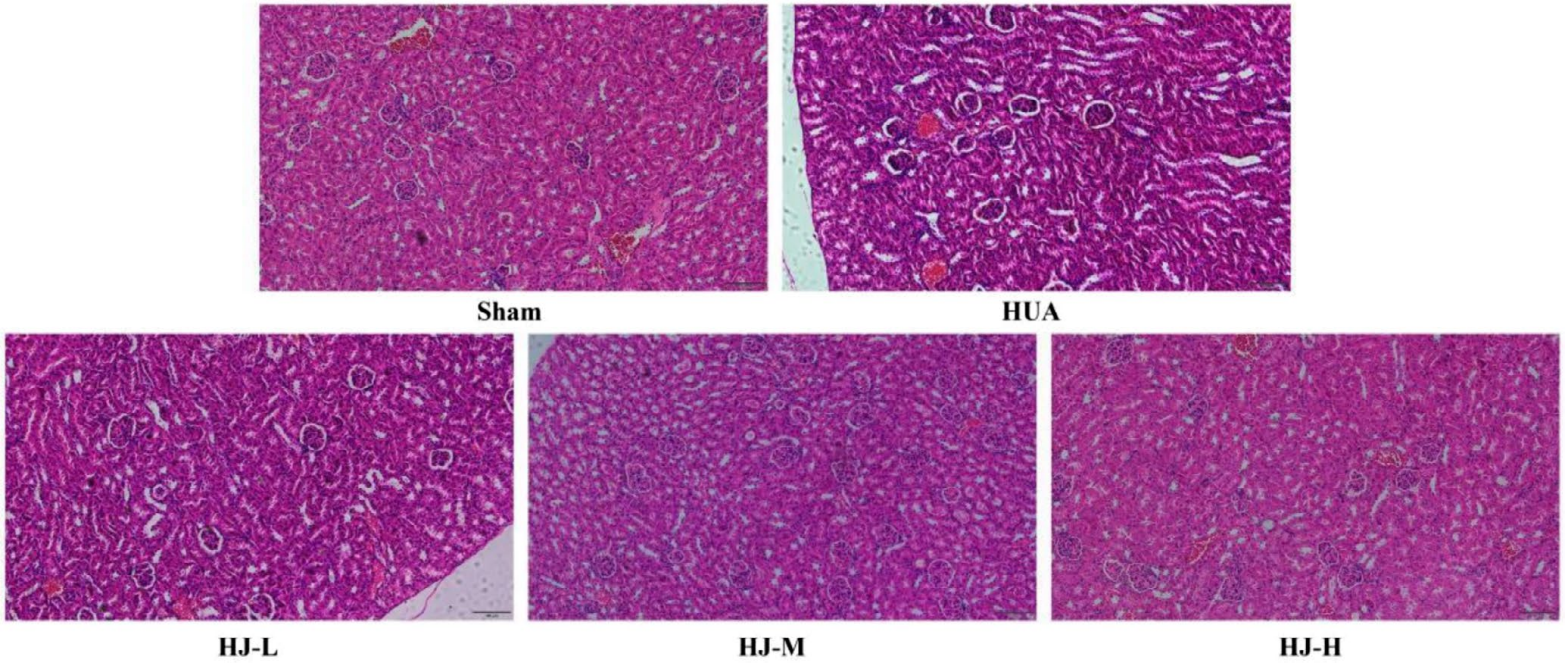
H&E staining results showed that HJ reduced renal tissue damage in HUA mice (*n* = 6).

As shown in Figure 3, H&E staining revealed that renal tissue from mice in the sham group exhibited well‐organized cellular architecture, with distinct and intact glomerular and tubular structures, uniform staining of tubular epithelial cells, and normal morphology. In contrast, the renal tissues of mice in the HUA group displayed glomerular deformation, atrophy, dilated renal tubules, and numerous vacuolar changes in the renal cells. Mice in the low‐dose HJ (HJ‐L) group showed kidney damage similar to that observed in the HUA group. However, mice in the HJ‐M and HJ‐H groups exhibited well‐preserved renal tissue with neatly arranged cells, clear and intact glomerular and tubular structures, uniform staining of tubular epithelial cells, and normal morphology.

**FIGURE 4:**
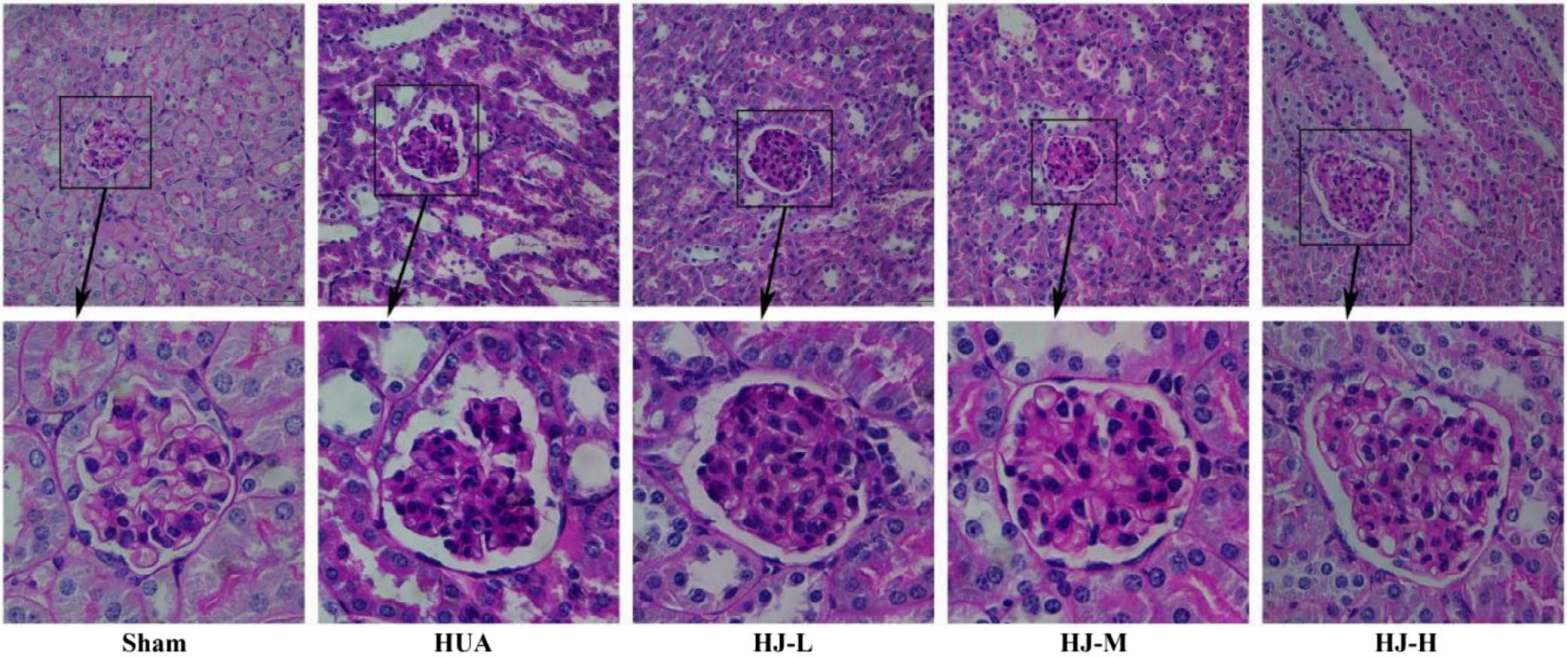
PAS staining results showed that HJ alleviated glomerular injury in HUA mice (*n* = 6).

Figure 4 illustrates the results of PAS staining, which revealed that the glomerular structures of the sham group mice were clear, full, and displayed normal morphology. In the HUA group, glomeruli exhibited basement membrane thickening. While the HJ‐L group also showed basement membrane thickening in the glomeruli, the HJ‐M and HJ‐H groups exhibited clear and well‐defined glomerular structures with normal morphology.

### 3.3 Results of Network Pharmacology Analysis

**FIGURE 5:**
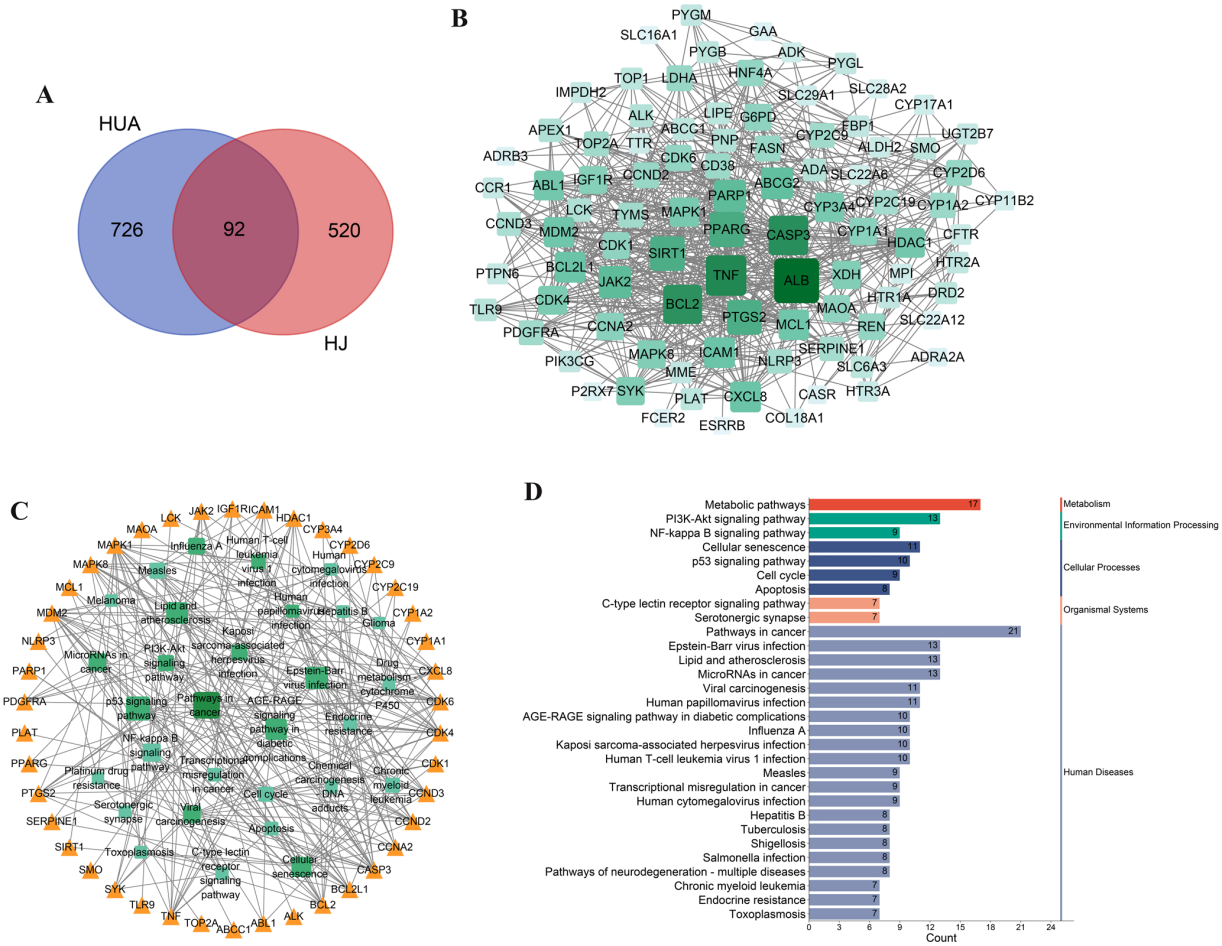
The results of network pharmacology analysis indicate thatHJ may treat HUA through metabolic pathways. (A) Venn analysis between HJ related targets and HUA related targets, where the intersection represents the effective targets of HJ after HUA treatment. (B) PPI network. In the network, dark green purple nodes can be considered important, while light green nodes are less important, and the width of the edges indicates the strength of protein connections. (C) Compounds‐targets network. The yellow triangle nodes represent targets, and the green square nodes represent compounds. The darker the color, the larger the nodes, indicating a higher degree value. (D) Top 30 clustering bar charts of KEGG pathway, with the first being metabolic pathways.

This study employed network pharmacology techniques to preliminarily analyze the possible mechanism by which HJ prevents and treats HUA. Data from the Integrative Pharmacology‐based Research Platform of TCMIP and TCMSP indicated that HJ may contain 43 compounds (Table S1). A total of 818 HUA‐related genes and 612 HJ targets were predicted from four databases: GeneCards, OMIM, DrugBank, and TTD (Data S1 and S2). Venn analysis identified 92 common targets of HJ and HUA (Figure 5A and Data S3). These 92 intersection targets were imported into the STRING database for protein interaction analysis, and the network was analyzed using Cytoscape software to obtain the PPI network (Figure 5B). Based on the PPI analysis results, 60 targets with a Degree ≥ 10 in the network were selected as the core interaction targets (Table S2).

Perform GO analysis on these 60 core targets. GO enrichment analysis was conducted using the DAVID database, and the results included three items: biological process, cellular component, and molecular function. According to the ascending order of *p*‐values, select the top 10 most significant terms for each item and plot them, as shown in Figure S1. KEGG analysis was performed on these 60 core targets, and the results are shown in Figure 5C and Data S4. Sort in ascending order of *p*‐values and select the top 30 most significant pathways for pathway target network analysis, as shown in Figure 3D. The KEGG analysis results showed that HJ may act on 17 targets including PYGB, G6PD, and MAOA, and treat HUA through metabolic pathways.

### 3.4 Effect of HJ on the Metabolic Levels of HUA Mice

To further investigate the potential metabolic pathways through which HJ exerts its therapeutic effects on HUA, untargeted metabolomics analysis was performed on the serum samples from mice in each experimental group. Given that the high‐dose HJ (HJ‐H) treatment group demonstrated the most significant therapeutic effect, metabolomics analysis was conducted on the sham, HUA, and HJ‐H groups.

**FIGURE 6:**
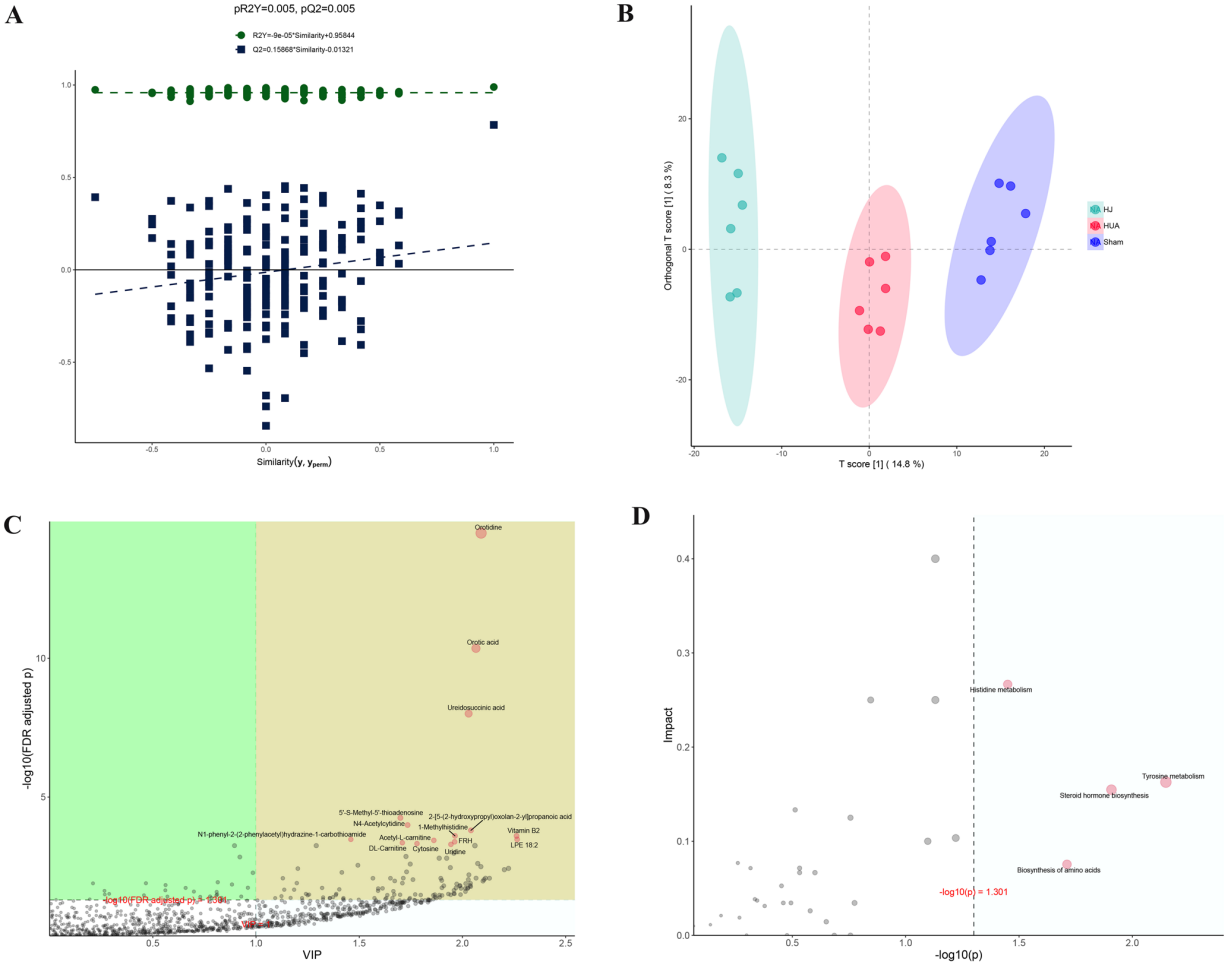
The results of untargeted metabolomics analysis indicatethat HJ may alleviate HUA through histidine metabolism, tyrosine metabolism, steroid hormone biosynthesis, and biosynthesis of amino acids. (A) PLS‐DA permutation test analysis chart. If pR2Y and pQ2 are less than 0.05, it indicates that the model is relatively reliable. (B) PLS‐DA point cloud map. The point cloud distribution of the three groups of samples is in different regions, indicating that the PLS‐DA model has a good discriminative effect. (C) PLS‐DA metabolite importance map. The metabolites with metabolite names marked in the yellow area are metabolites with *p*‐values less than 0.05 and VIP greater than 1 after correction. These metabolites have significant differences between groups and play an important role in PLS‐DA, which should be given special attention. (D) ORA enrichment analysis and topological analysis. The metabolic pathway of pink dots is a significant metabolic pathway in ORA enrichment analysis.

Partial least squares discriminant analysis (PLS‐DA) of the serum metabolites from each group was performed. The results of the PLS‐DA permutation test indicated that both pR2Y and pQ2 values were less than 0.05, suggesting that the model is robust and reliable (Figure 6A). The PLS‐DA score plot revealed clear separation of the point clouds between the groups, indicating substantial differences in the metabolite profiles and structures across the groups (Figure 6B).

Metabolites were selected based on the variable importance in the projection (VIP) score, with a threshold of VIP > 1, and a *p*‐value cutoff of 0.05 for statistical significance. Using these criteria, the top 15 metabolites with significant differences were identified and selected through secondary mass spectrometry (Figure 6C and Figure S2). The analysis revealed that HUA induced alterations in the levels of certain metabolites, which were reversed by HJ treatment.

To explore the underlying biological pathways, MetaboAnalyst 5.0 (https://www.metaboanalyst.ca/) was used for pathway enrichment analysis of the differentially abundant metabolites, as shown in Figure 6D. The results indicated that HJ significantly influenced key metabolic pathways in the serum of HUA mice, including tyrosine metabolism, steroid hormone biosynthesis, biosynthesis of amino acids, and histidine metabolism (Figures S3–S7).

Further analysis of the relationships between 17 core targets and these four metabolic pathways revealed that MAOA is involved in histidine metabolism, while CYP3A4, CYP1A2, and CYP1A1 play roles in steroid hormone biosynthesis (Figures S8 and S9).

### 3.5 Effect of HJ on Protein Expression of MAOA, CYP3A4, CYP1A2 and CYP1A1

**FIGURE 7:**
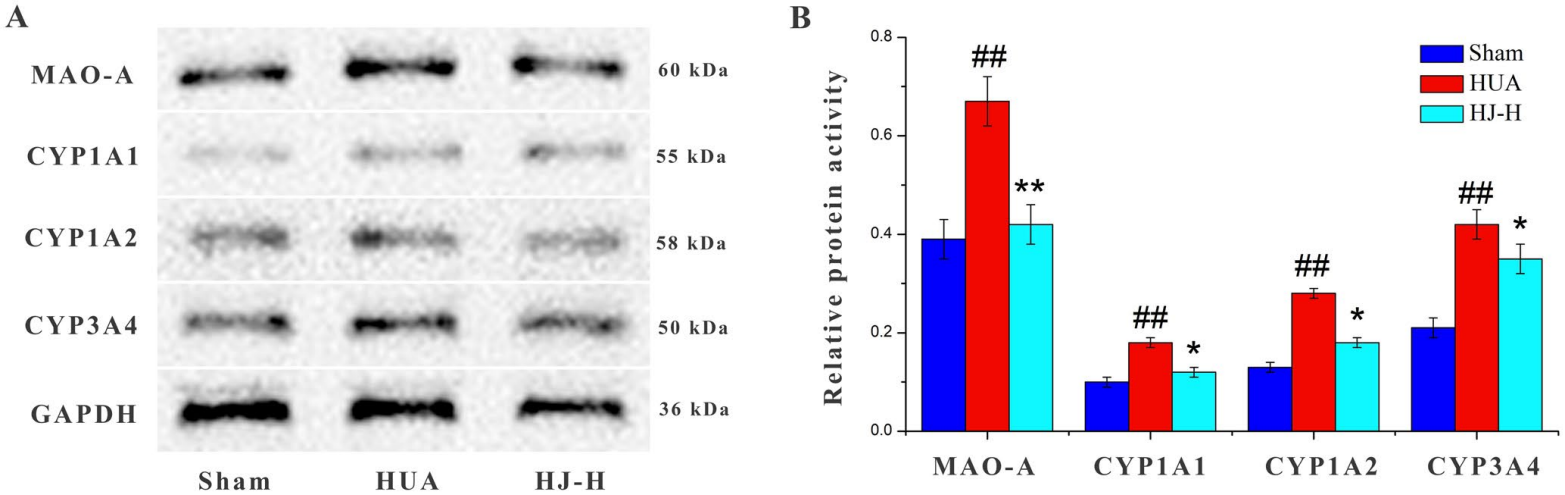
The effect of HJ on the protein levels of MAO‐A,CYP1A1, CYP1A2, and CYP3A4 in mice liver tissue. (A) Measurement of MAO‐A, CYP1A1, CYP1A2, and CYP3A4 protein levels in rat hearts by western blot (*n* = 3). (B) Quantitative analysis of MAO‐A, CYP1A1, CYP1A2, and CYP3A4 protein levels. Data are presented as mean ± SD. ^##^ *p* < 0.01 compared to the sham group; *p < 0.05, **p < 0.01 compared to the HUA group.

Western blotting was employed to evaluate the protein expression levels of MAOA, CYP3A4, CYP1A2, and CYP1A1 in the liver tissues of mice from the sham, HUA, and HJ‐H groups. As shown in Figure 7, the protein expression of MAOA, CYP3A4, CYP1A2, and CYP1A1 was significantly elevated in the liver tissue of the HUA group compared to the sham group. However, in the HJ‐H treatment group, the protein levels of MAOA, CYP3A4, CYP1A2, and CYP1A1 were significantly reduced compared to the HUA group.

### 3.6 HJ Reduces HA Levels in HUA Patients

**FIGURE 8:**
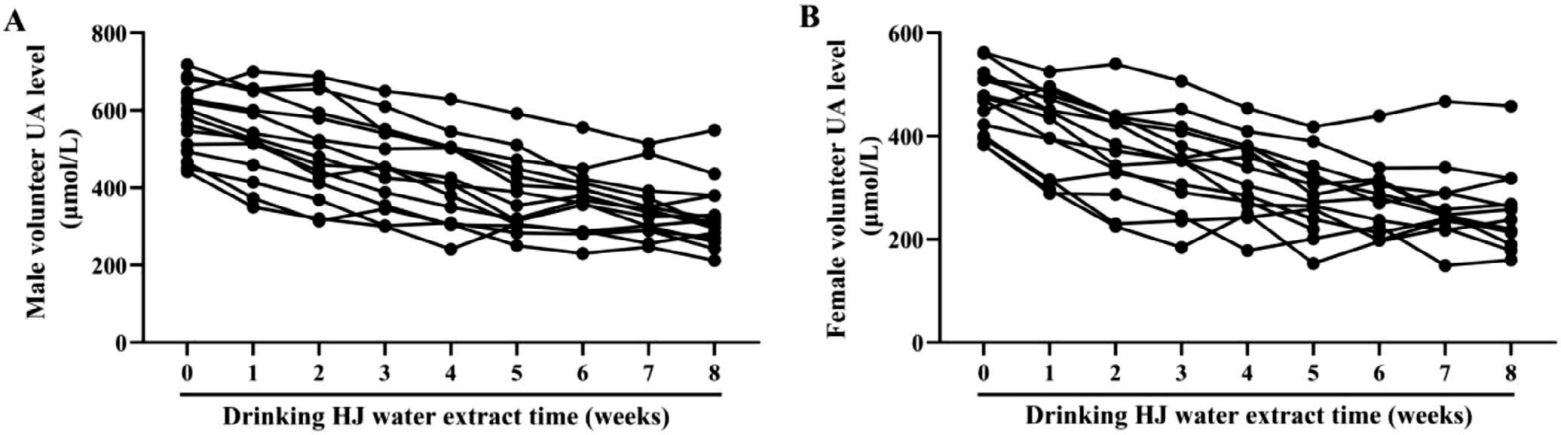
HJ reduces UA levels in male (A) and female (B) volunteers.

A total of 30 volunteers with HUA were enrolled for this study, comprising 15 males and 15 females. After treatment with HJ, all volunteers exhibited a decrease in serum UA levels (Figure 8). These results suggest that HJ is effective in reducing UA levels in individuals with HUA.

## 4 Discussion

HUA is a chronic metabolic disease caused by purine metabolism disorders, mainly due to reduced or increased excretion of UA (James et al. 2023). In addition, consuming excessive foods high in purine may also trigger the onset of HUA (Wen et al. 2024). This study used a specially designed feed 2% (w/w) UA and 3% (w/w) potassium oxonate to construct a mice HUA model. The results showed that the levels of UA, Cr, and BUN in the serum of the modeling mice were significantly increased. These results indicate that the HUA modeling in this study was successful. After administering HJ to HUA mice, the levels of UA, Cr, and BUN in the serum of HUA mice were significantly reduced.

Renal damage is a common complication of HUA (Guo et al. 2023). Our study revealed severe damage to both the kidneys and glomeruli of HUA mice, which was significantly mitigated by HJ treatment. Importantly, we observed that HJ not only ameliorated HUA in mice but also effectively reduced serum UA levels in human patients with HUA. These findings collectively indicate that HJ exhibits therapeutic potential for HUA treatment.

Network pharmacology, based on systems biology theory, is a widely used method for investigating the mechanisms of traditional Chinese medicine through biological network analysis (Zhao et al. 2023; Zhang et al. 2021).

This approach provides a comprehensive framework for understanding complex herb‐disease interactions. In this study, we employed network pharmacology to elucidate the potential mechanisms by which HJ treats HUA. The results revealed that HJ may exert its therapeutic effects on HUA by modulating multiple targets, including PYGB, G6PD, MAOA, TYMS, CYP3A4, PTGS2, CYP2C19, SIRT1, CYP2C9, LDHA, PNP, FASN, CYP1A2, CYP1A1, CD38, XDH, and ADA, which collectively regulate metabolic pathways implicated in HUA pathogenesis. This multi‐target approach is characteristic of herbal medicines and may contribute to HJ's efficacy in treating HUA.

To further investigate whether HJ treats HUA through metabolic pathways, we conducted untargeted metabolomics analysis. This powerful analytical approach allows for the comprehensive profiling of metabolites, providing insights into the biochemical changes induced by HJ treatment. The results indicated that HJ may ameliorate HUA by regulating tyrosine metabolism, steroid hormone biosynthesis, biosynthesis of amino acids, and histidine metabolism.

We then performed an in‐depth analysis to elucidate the relationship between the 17 core targets identified through network pharmacology and the four key metabolic pathways revealed by metabolomics. The results demonstrated that MAOA participates in histidine metabolism, while CYP3A4, CYP1A2, and CYP1A1 are involved in steroid hormone biosynthesis. This integration of network pharmacology and metabolomics findings provides a more comprehensive understanding of HJ's mechanism of action.

To validate these findings at the protein level, we examined the expression of MAOA, CYP3A4, CYP1A2, and CYP1A1 in the liver tissue of HUA mice. The results showed significantly increased protein expression of these targets in HUA mice compared to controls. Notably, HJ treatment significantly reduced the protein expression of MAOA, CYP3A4, CYP1A2, and CYP1A1. These findings corroborate the results from our network pharmacology and metabolomics analyses, providing further evidence for HJ's mechanism of action.

These results collectively suggest that HJ may exert its therapeutic effects on HUA through dual mechanisms: modulation of histidine metabolism via MAOA targets and regulation of steroid hormone biosynthesis via CYP3A4, CYP1A2, and CYP1A1 targets. This multi‐faceted approach may contribute to HJ's efficacy in treating HUA and highlights the complex nature of herbal medicine actions.

## 5 Conclusion

In conclusion, this study demonstrates that HJ exhibits significant therapeutic potential for managing HUA, primarily through mechanisms involving the regulation of histidine metabolism via MAOA targets and the modulation of steroid hormone biosynthesis via CYP3A4, CYP1A2, and CYP1A1 targets. Our integrated approach, combining network pharmacology, metabolomics, and protein expression analysis, provides a comprehensive understanding of HJ's mechanism of action in treating HUA.

As a food‐medicine homologous substance, HJ offers notable advantages, including exceptionally low toxicity and a superior safety profile compared to conventional pharmaceutical treatments. This favorable safety profile, combined with its demonstrated efficacy, positions HJ as a promising candidate for long‐term HUA management. Thus, HJ represents a promising therapeutic strategy for the prevention and management of HUA, particularly when developed into functional food formulations.

In future research, we aim to develop HJ into various functional food products with the ability to prevent and treat HUA, such as biscuits, jellies, and beverages. This approach could provide patients with convenient and palatable options for managing their condition, potentially improving compliance and long‐term outcomes.

### 5.1 Limitations

This study has several important limitations that warrant acknowledgment. First, the HJ water extract is a complex mixture, and the specific active components responsible for its anti‐HUA effects remain unidentified. Future studies should focus on isolating and characterizing these active compounds to better understand their individual contributions to HJ's therapeutic effects. Second, comprehensive mechanistic studies exploring the anti‐HUA effects of HJ are lacking. While our integrated approach provides valuable insights, further in‐depth investigations, including gene knockout studies and pathway inhibition experiments, are needed to fully elucidate the molecular mechanisms underlying HJ's effects. Third, while this study demonstrated a reduction in UA levels among HUA volunteers following HJ consumption, the absence of a rigorously designed control group precludes definitive conclusions about HJ's anti‐HUA efficacy. Future clinical trials should include a placebo‐controlled, double‐blind design with a larger sample size to more robustly evaluate HJ's efficacy in treating HUA. These limitations will be systematically addressed in future research endeavors to further validate and expand upon the findings presented in this study.

## Author Contributions

**Peng Zhao:** conceptualization (equal), data curation (equal), writing – original draft (equal). **Wenjing Liu:** data curation (equal), formal analysis (equal). **Minyang He:** writing – original draft (equal). **Weiyou Cao:** investigation (equal), software (equal). **Qichang Xu:** software (equal). **Qing Hao:** investigation (equal), methodology (equal). **Lijun Wang:** investigation (equal), software (equal). **Ying Liu:** formal analysis (equal), investigation (equal). **Jingyu Wang:** investigation (equal), methodology (equal). **Feixue Wang:** investigation (equal), methodology (equal). **Lin Jiang:** formal analysis (equal), investigation (equal). **Songze Li:** investigation (equal), software (equal). **Chunyan Dai:** software (equal), visualization (equal). **Qiusheng Zheng:** supervision (equal), validation (equal). **Jun Ma:** investigation (equal), methodology (equal). **Xiangcheng Fan:** writing – original draft (equal), writing – review and editing (equal). **Jichun Han:** funding acquisition (equal), resources (equal), writing – original draft (equal), writing – review and editing (equal).

## Ethics Statement

The animal use protocol listed below was reviewed and approved by the Binzhou Medical University Institutional Animal Care and Use Committee (2023‐378).

## Conflicts of Interest

The authors declare no conflicts of interest.

## Supporting information


**Data S1:** fsn370985‐sup‐0001‐Supplementary 1.xlsx.

**Data S2:** fsn370985‐sup‐0002‐Supplementary 2.xlsx.

**Data S3:** fsn370985‐sup‐0003‐Supplementary 3.xlsx.

**Data S4:** fsn370985‐sup‐0004‐Supplementary 4.xlsx.

**Appendix S1:** fsn370985‐sup‐0005‐Supinfo.docx.

## Data Availability

The raw datasets used and analyzed during the study are available on request to the senior author (J.H., 923023681@qq.com).
