# Supplementary material for: Polygonatum sibiricum Delar. ex Redoute: A Potential Functional Food for the Prevention and Treatment of Hyperuricemia
Source: Food Sci Nutr. 2025 Oct 11;13(10):e70985. doi: 10.1002/fsn3.70985 (PMC12514339; doi:10.1002/fsn3.70985)
Supplement: Supplementary file 5 — Appendix S1: fsn370985‐sup‐0005‐Supinfo.docx. [file FSN3-13-e70985-s001.docx]

**Supporting Information for**

***Polygonatum sibiricum* Delar. ex Redoute can be prepared as a food with the ability to prevent and treat hyperuricaemia**

Peng Zhao ^2#^, Wenjing Liu ^1#^, Minyang He^4#^, Weiyou Cao ^1^, Qichang Xu ^1^, Qing Hao ^1^, Lijun Wang ^1^, Ying Liu ^1^, Jingyu Wang ^1^, Feixue Wang ^1^, Lin Jiang ^1^, Songze Li ^1^, Chunyan Dai^5^, Qiusheng Zheng ^1^, Jun Ma ^1^, Xiangcheng Fan ^3,6*^, Jichun Han ^1*^

*^1^College of Traditional Chinese Medicine, Binzhou Medical University, Yantai, China, 264003*

*^2^Department of Cardiac Surgery I, Yantai Yuhuangding Hospital, Yantai, China, 264099*

*^3^Department of Pharmacy, Center for Membrane Receptor and Brain Medicine, the Fourth Affiliated Hospital of School of Medicine, and International School of Medicine, International Institutes of Medicine, Zhejiang University, Yiwu, China, 322000.*

*^4^Department of Gastroenterology, the Fourth Affiliated Hospital of School of Medicine, and International School of Medicine, International Institutes of Medicine, Zhejiang University, Yiwu,China,322000*

*^5^Department of Pathology, The Fourth Affiliated Hospital of School of Medicine, and International School of Medicine, International Institutes of Medicine, Zhejiang*

*University, Yiwu, 322000, China*

*^6^Center for Innovative Traditional Chinese Medicine Target and New Drug Research, International Institutes of Medicine, Zhejiang University, Yiwu, China, 322000*

^#^These authors contributed equally.

^*^Correspondence: [923023681@qq.com](mailto:923023681@qq.com) (J.H) and xcfan@zju.edu.cn (X.F)

**Supplementary Materials**

**
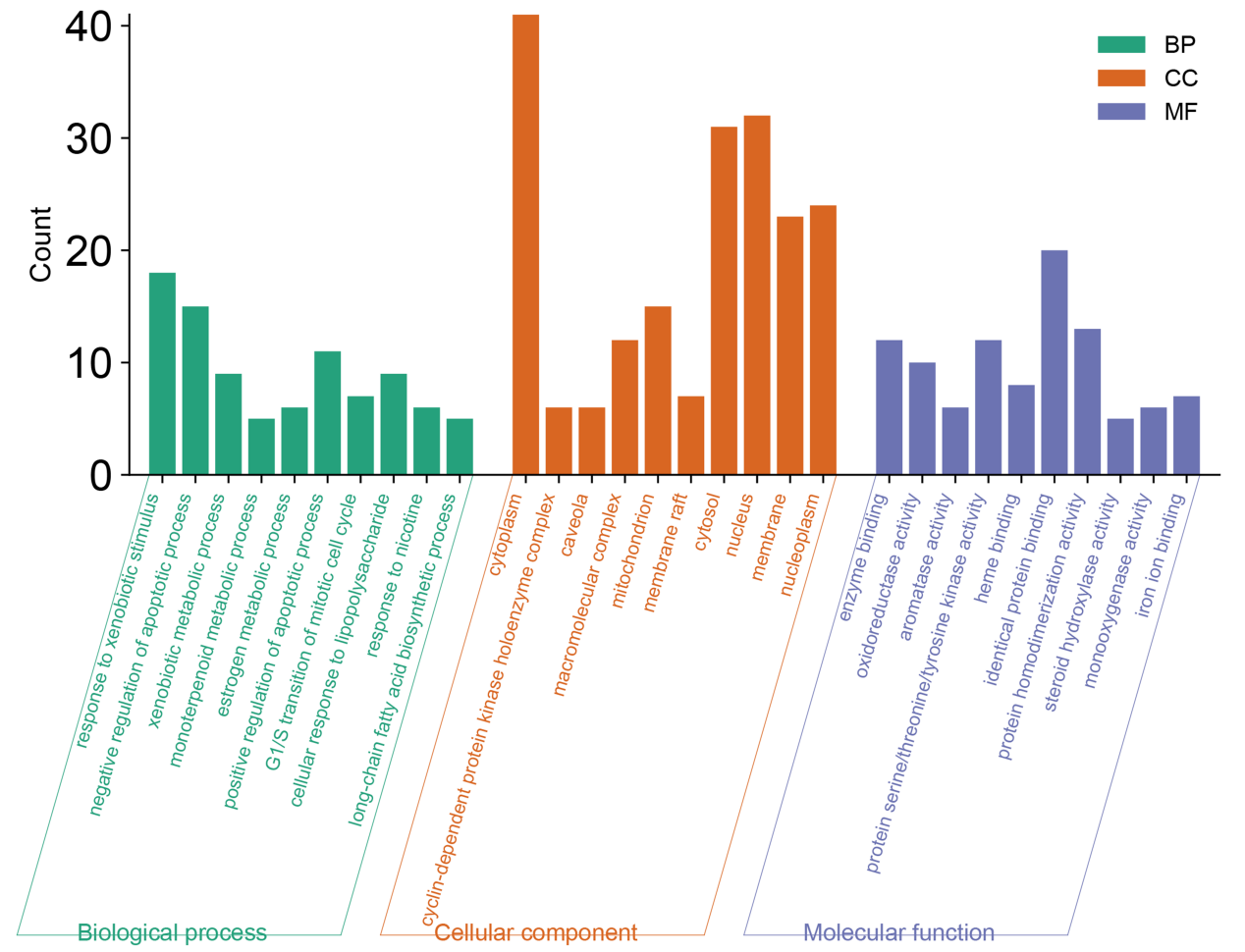
**

**Figure S1.** Gene Ontology (GO) analysis of the core targets identified through network pharmacology. The top 10 most significant biological processes (BP), cellular components (CC), and molecular functions (MF) are presented based on the GO enrichment analysis of the 60 core targets identified in the PPI network.

**
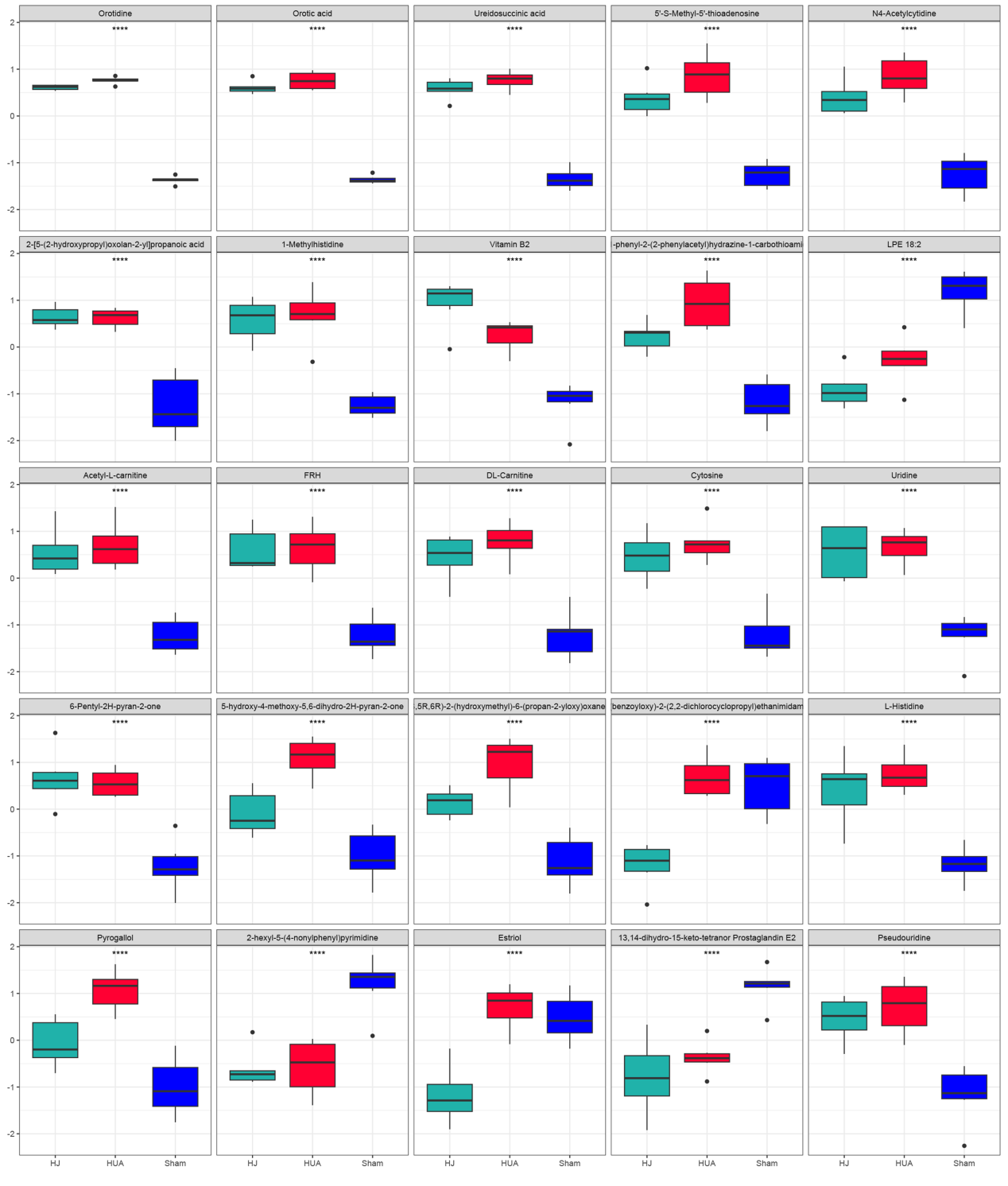
**

**Figure S2.** Significant features identified through untargeted metabolomics analysis. The figure shows the metabolites with significant differences between the treatment groups based on the PLS-DA model, with a VIP score > 1 and p-value < 0.05.

**
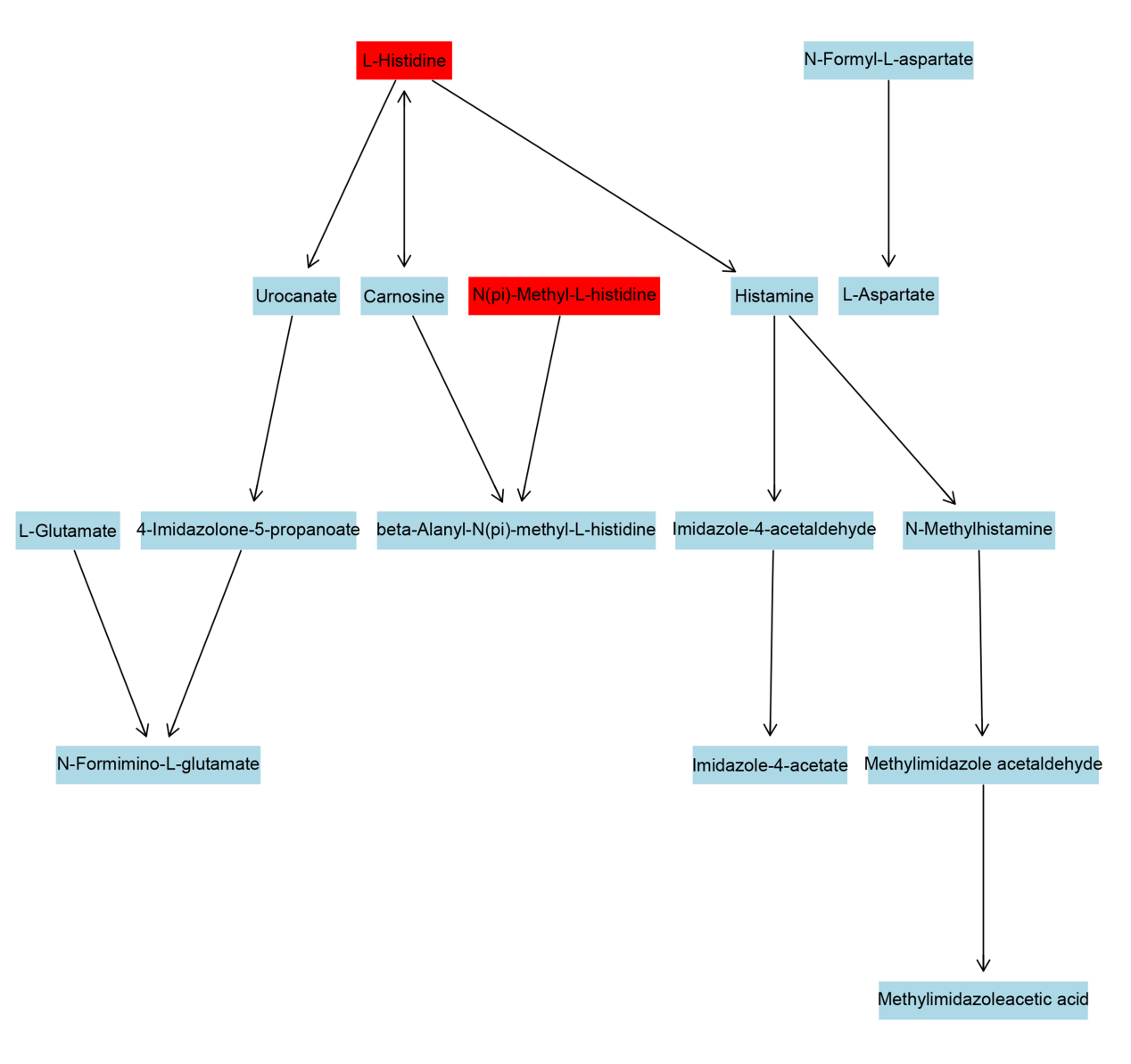
**

**Figure S3.** Histidine metabolism pathway analysis in HUA mice. The altered levels of metabolites in the histidine metabolism pathway in the serum of HUA mice after HJ treatment are shown, as identified through metabolomics analysis.

**
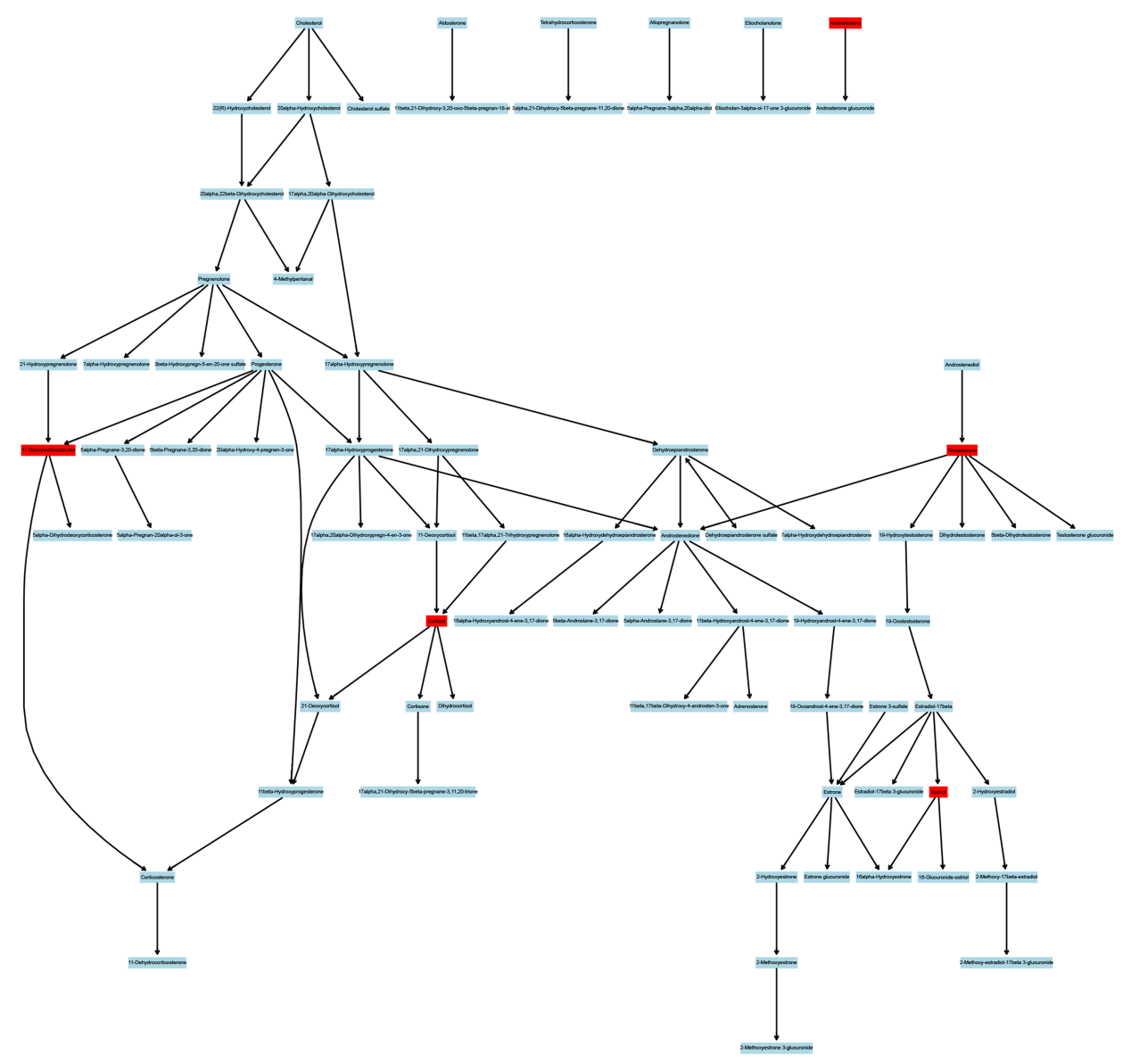
**

**Figure S4.** Steroid hormone biosynthesis pathway analysis in HUA mice. Metabolomics analysis reveals the significant changes in metabolites involved in steroid hormone biosynthesis, highlighting the effects of HJ treatment on this pathway.

**
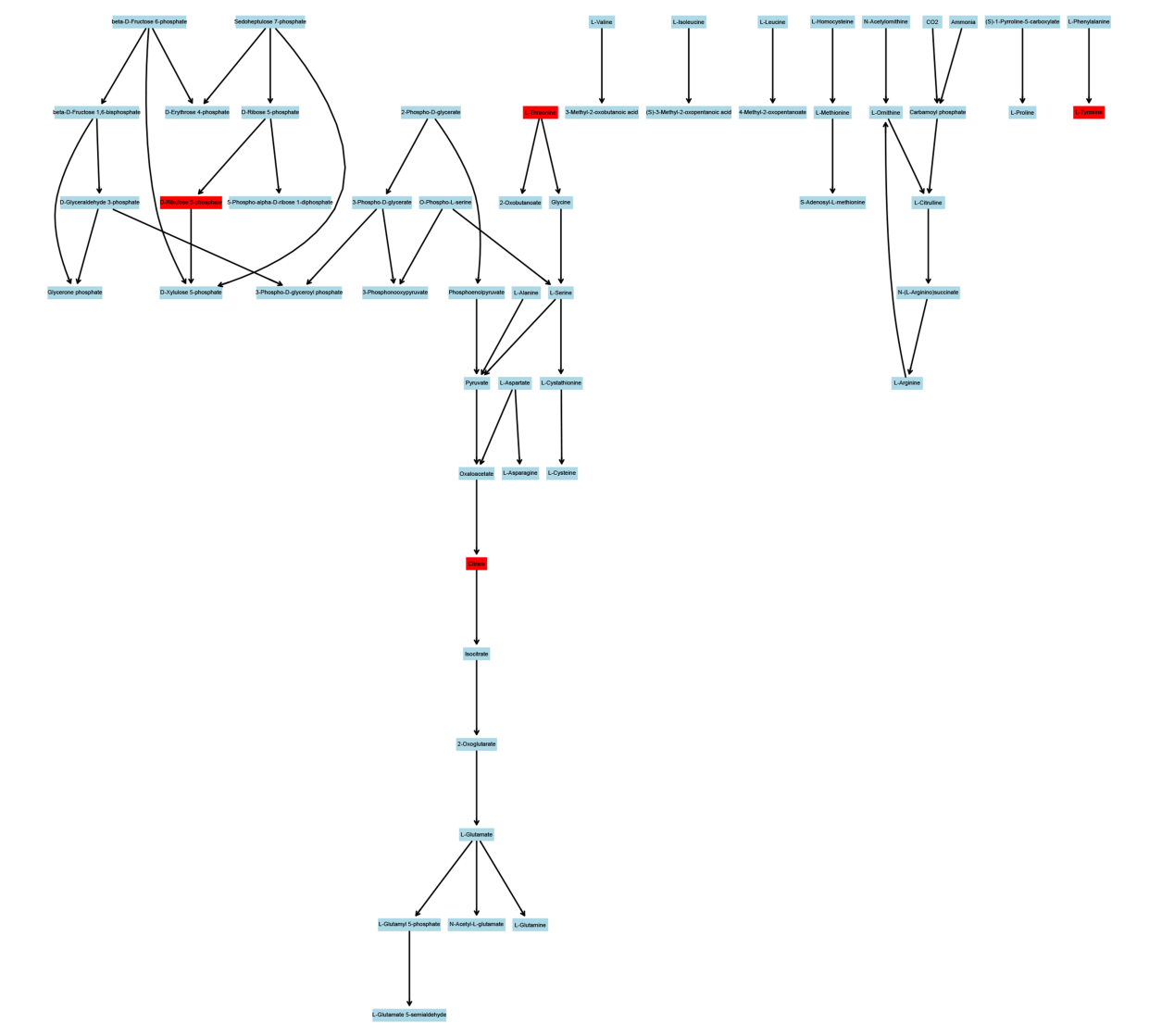
**

**Figure S5.** Biosynthesis of amino acids pathway analysis in HUA mice. This figure depicts the metabolites related to amino acid biosynthesis and their significant changes in the serum of HUA mice treated with HJ.

**
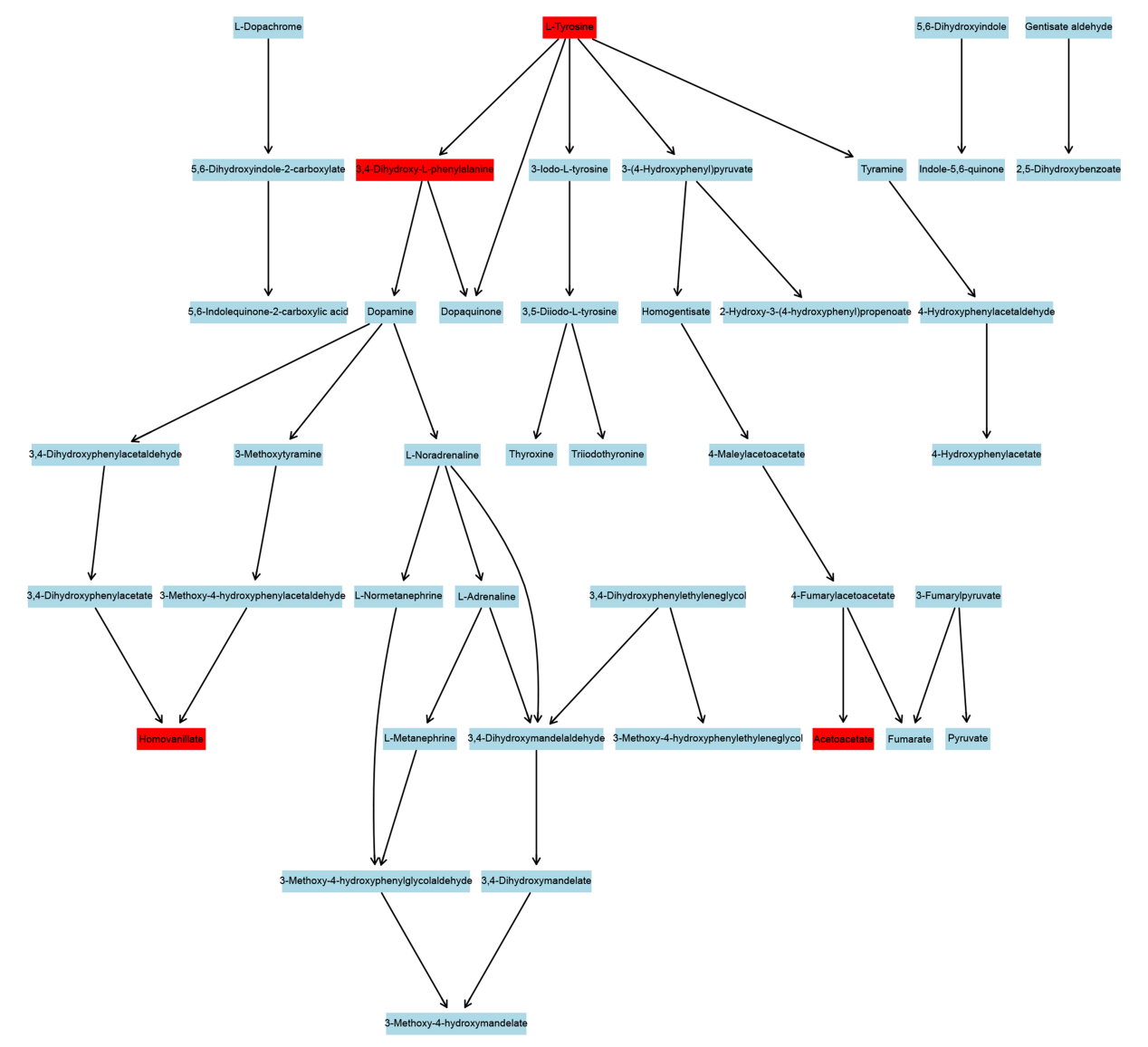
**

**Figure S6.** Tyrosine metabolism pathway analysis in HUA mice. The alterations in metabolites associated with tyrosine metabolism in the serum of HUA mice following HJ treatment are illustrated here.

**
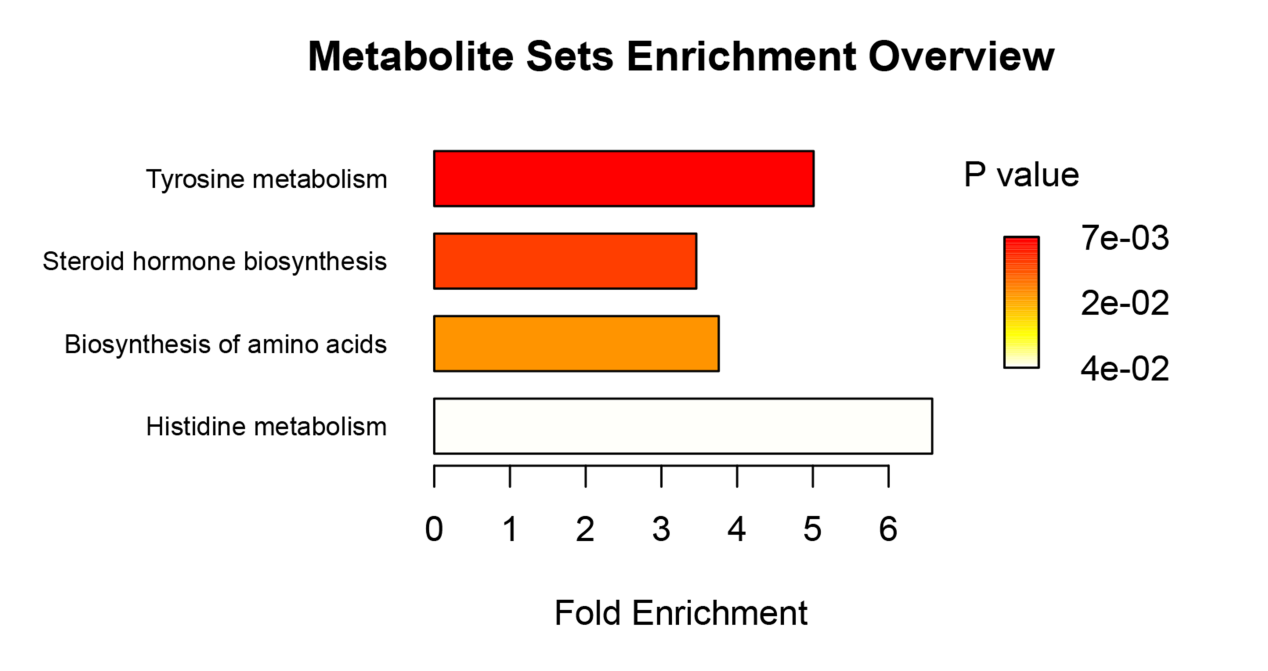
**

**Figure S7.** ORA hyper score for pathway enrichment. The figure displays the results of the overrepresentation analysis (ORA) of the metabolic pathways influenced by HJ treatment, with the top pathways showing significant enrichment based on p-values.

**
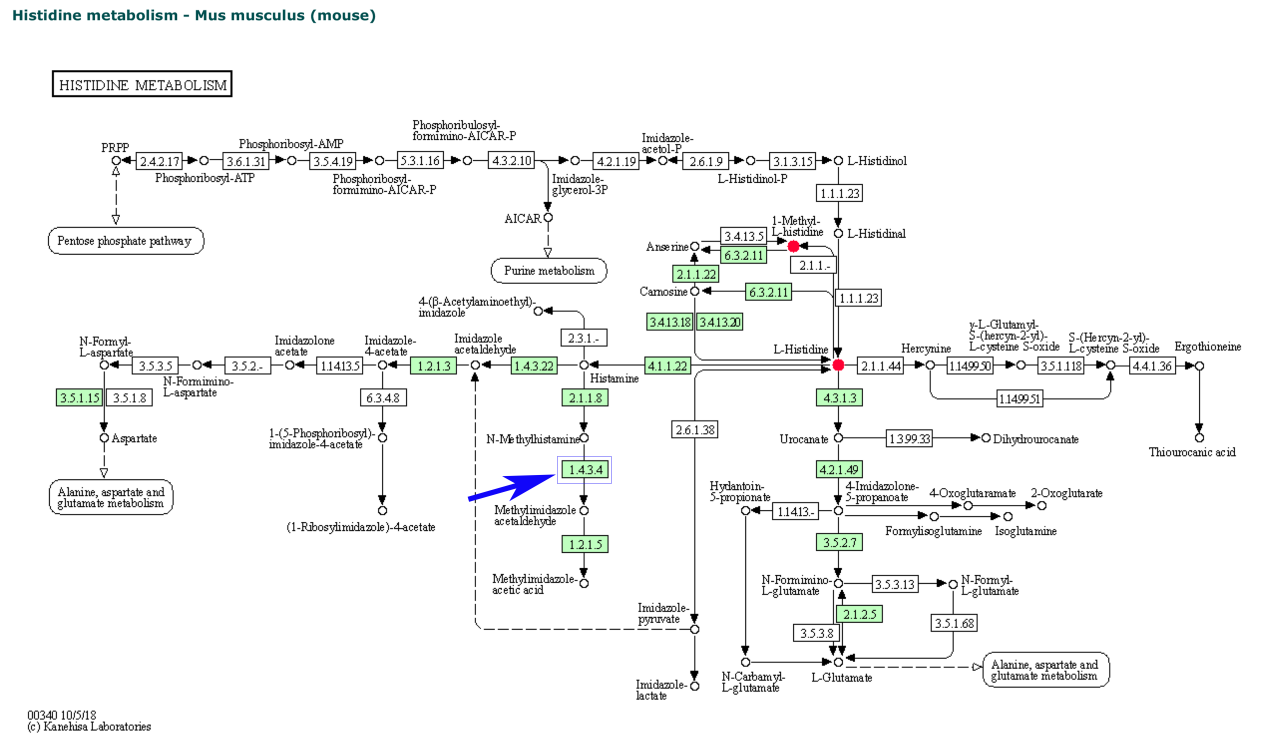
**

**Figure S8.** KEGG pathway analysis for histidine metabolism in *Mus musculus* (mouse). This figure highlights the histidine metabolism pathway and its alterations in response to HJ treatment, as determined by KEGG pathway enrichment analysis.

**
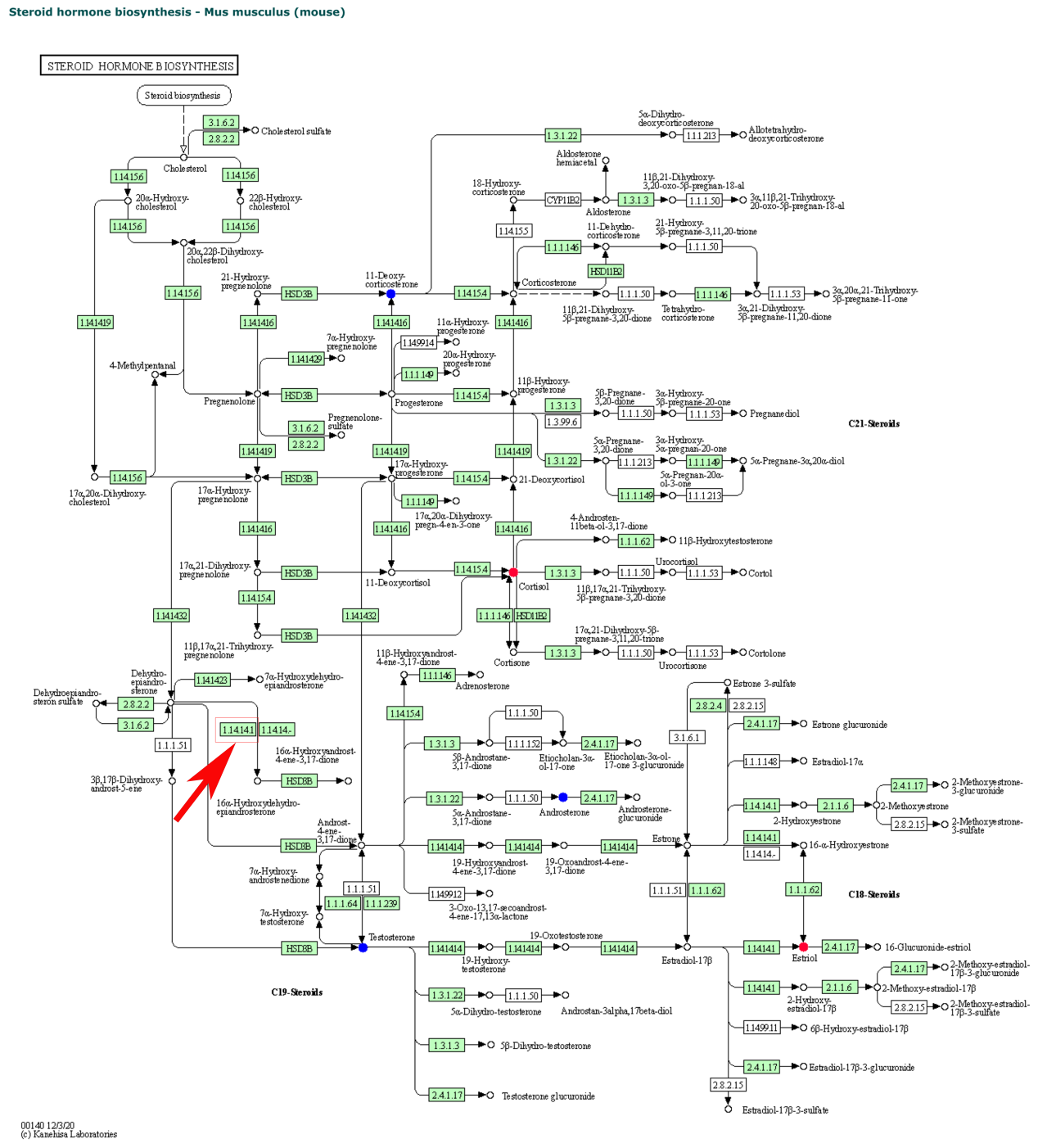
**

**Figure S9.** KEGG pathway analysis for steroid hormone biosynthesis in *Mus musculus* (mouse). The figure shows the steroid hormone biosynthesis pathway and its alterations following HJ treatment, identified through KEGG pathway enrichment analysis.

**Table S1** Compounds contained in HJ and their corresponding IUPAC names. This table lists 43 active compounds identified in HJ, including their chemical names and structures, as determined through network pharmacology analysis.

| **Number** | **Compound** | **IUPAC Name** |
| --- | --- | --- |
| 1 | isoliquiritigenin | (E)-1-(2,4-dihydroxyphenyl)-3-(4-hydroxyphenyl)prop-2-en-1-one |
| 2 | DFV | (S)-7-hydroxy-2-(4-hydroxyphenyl)chroman-4-one |
| 3 | salicylic acid | 2-hydroxybenzoic acid |
| 4 | baicalein | 5,6,7-trihydroxy-2-phenyl-4H-chromen-4-one |
| 5 | 3'-Methoxydaidzein | 7-hydroxy-3-(4-hydroxy-3-methoxyphenyl)-4H-chromen-4-one |
| 6 | succinic acid | [succinic acid](https://old.tcmsp-e.com/molecule.php?qn=346) |
| 7 | Sitogluside | (2R,3R,4S,5S,6R)-2-(((3S,8S,9S,10R,14R,17R)-17-((2R,5R)-5-ethyl-6-methylheptan-2-yl)-10-methyl-2,3,4,7,8,9,10,11,12,13,14,15,16,17-tetradecahydro-1H-cyclopenta[a]phenanthren-3-yl)oxy)-6-(hydroxymethyl)tetrahydro-2H-pyran-3,4,5-triol |
| 8 | beta-sitosterol | (3S,8S,9S,10R,13R,14S,17R)-17-((2R,5R)-5-ethyl-6-methylheptan-2-yl)-10,13-dimethyl-2,3,4,7,8,9,10,11,12,13,14,15,16,17-tetradecahydro-1H-cyclopenta[a]phenanthren-3-ol |
| 9 | sitosterol | (3R,8S,9S,10R,13R,14S,17R)-17-((2R,5R)-5-ethyl-6-methylheptan-2-yl)-10,13-dimethyl-2,3,4,7,8,9,10,11,12,13,14,15,16,17-tetradecahydro-1H-cyclopenta[a]phenanthren-3-ol |
| 10 | methylprotodioscin | (2S,3R,4R,5S,6S)-2-(((2S,3S,4R,5R,6S)-4-hydroxy-2-(hydroxymethyl)-6-(((4S,6aR,6bR,8bS,9R,10R,11aR)-10-methoxy-6a,8a,9-trimethyl-10-((S)-3-methyl-4-(((2S,3R,4R,5S,6S)-3,4,5-trihydroxy-6-(hydroxymethyl)tetrahydro-2H-pyran-2-yl)oxy)butyl)-3,4,5,6,6a,6b,7,8,8a,8b,9,10,11a,12,12a,12b-hexadecahydro-1H-naphtho[2',1':4,5]indeno[2,1-b]furan-4-yl)oxy)-5-(((2R,3R,4R,5S,6S)-3,4,5-trihydroxy-6-methyltetrahydro-2H-pyran-2-yl)oxy)tetrahydro-2H-pyran-3-yl)oxy)-6-methyltetrahydro-2H-pyran-3,4,5-triol |
| 11 | methylprotodioscin_qt | (4S,6aR,6bR,8bS,9R,10R,11aR)-10-((S)-4-hydroxy-3-methylbutyl)-10-methoxy-6a,8a,9-trimethyl-3,4,5,6,6a,6b,7,8,8a,8b,9,10,11a,12,12a,12b-hexadecahydro-1H-naphtho[2',1':4,5]indeno[2,1-b]furan-4-ol |
| 12 | (+)-Syringaresinol | 4,4'-((1S,3aR,4S,6aR)-tetrahydro-1H,3H-furo[3,4-c]furan-1,4-diyl)bis(2,6-dimethoxyphenol) |
| 13 | (Z)-1-(2,4-dihydroxyphenyl)-3-(4-hydroxyphenyl)prop-2-en-1-one | (Z)-1-(2,4-dihydroxyphenyl)-3-(4-hydroxyphenyl)prop-2-en-1-one |
| 14 | glucuronic acid | (2S,3S,4S,5R,6R)-3,4,5,6-tetrahydroxytetrahydro-2H-pyran-2-carboxylic acid |
| 15 | neoliquiritin | (R)-2-(4-hydroxyphenyl)-7-(((2S,3R,4S,5S,6R)-3,4,5-trihydroxy-6-(hydroxymethyl)tetrahydro-2H-pyran-2-yl)oxy)chroman-4-one |
| 16 | (2R)-7-hydroxy-2-(4-hydroxyphenyl)chroman-4-one | (R)-7-hydroxy-2-(4-hydroxyphenyl)chroman-4-one |
| 17 | GUP | (2R,3S,4S,5S,6R)-6-(hydroxymethyl)tetrahydro-2H-pyran-2,3,4,5-tetraol |
| 18 | BGC | (2R,3R,4R,5S,6R)-6-hydroperoxytetrahydro-2H-pyran-2,3,4,5-tetraol |
| 19 | diosgenin | (4S,5'R,6aR,6bS,8aS,8bR,9S,10R,11aS,12aS,12bS)-5',6a,8a,9-tetramethyl-1,3,3',4,4',5,5',6,6a,6b,6',7,8,8a,8b,9,11a,12,12a,12b-icosahydrospiro[naphtho[2',1':4,5]indeno[2,1-b]furan-10,2'-pyran]-4-ol |
| 20 | 4',5-Dihydroxyflavone | 5-hydroxy-2-(4-hydroxyphenyl)-4H-chromen-4-one |
| 21 | 4-[(1R,3aS,4R,6aS)-4-(4-hydroxy-3,5-dimethoxyphenyl)-1,3,3a,4,6,6a-hexahydrofuro[4,3-c]furan-1-yl]-2,6-dimethoxyphenol | 4,4'-((1R,3aS,4R,6aS)-tetrahydro-1H,3H-furo[3,4-c]furan-1,4-diyl)bis(2,6-dimethoxyphenol) |
| 22 | HMF | 5-(hydroxymethyl)furan-2-carbaldehyde |
| 23 | apigenin | 5,7-dihydroxy-2-(4-hydroxyphenyl)-4H-chromen-4-one |
| 24 | 2',7-dihydroxy-3',4'-dimethoxyisoflavane | 3-(2-hydroxy-4,5-dimethoxyphenyl)-2H-chromen-7-ol |
| 25 | 4-methylolfurfural | 4-(hydroxymethyl)furan-2-carbaldehyde |
| 26 | Oroxin A | 5,6-dihydroxy-2-phenyl-7-(((2S,3R,4S,5S,6R)-3,4,5-trihydroxy-6-(hydroxymethyl)tetrahydro-2H-pyran-2-yl)oxy)-4H-chromen-4-one |
| 27 | 2-Acridinecarboxylic acid | acridine-2-carboxylic acid |
| 28 | liriodendrin | (2R,2'R,3S,3'S,4S,4'S,5R,5'R,6S,6'S)-6,6'-((((1S,3aR,4S,6aR)-tetrahydro-1H,3H-furo[3,4-c]furan-1,4-diyl)bis(2,6-dimethoxy-4,1-phenylene))bis(oxy))bis(2-(hydroxymethyl)tetrahydro-2H-pyran-3,4,5-triol) |
| 29 | n-butyl-β-D-fructopyranoside | (2R,3S,4R,5S)-2-butoxy-2-(hydroxymethyl)tetrahydro-2H-pyran-3,4,5-triol |
| 30 | 2-Propen-1-one, 1-(2,4-dihydroxyphenyl)-3-(4-(beta-D-glucopyranosyloxy)phenyl)-, (2E)- | (E)-1-(2,4-dihydroxyphenyl)-3-(4-(((2S,3R,4S,5S,6R)-3,4,5-trihydroxy-6-(hydroxymethyl)tetrahydro-2H-pyran-2-yl)oxy)phenyl)prop-2-en-1-one |
| 31 | sibiricoside A | (2S,3S,4S,5R,6R)-2-(((2S,3S,4S,5S,6R)-2-(((2R,3R,4R,5S,6R)-4,5-dihydroxy-2-(hydroxymethyl)-6-(((4S,6aS,8aR,9S,10S,12aR,12bR)-10-methoxy-6a,8a,9-trimethyl-10-((R)-3-methyl-4-(((2R,3S,4S,5R,6R)-3,4,5-trihydroxy-6-(hydroxymethyl)tetrahydro-2H-pyran-2-yl)oxy)butyl)-3,4,5,6,6a,6b,7,8,8a,8b,9,10,11a,12,12a,12b-hexadecahydro-1H-naphtho[2',1':4,5]indeno[2,1-b]furan-4-yl)oxy)tetrahydro-2H-pyran-3-yl)oxy)-5-hydroxy-6-(hydroxymethyl)-4-(((2R,3R,4R,5R)-3,4,5-trihydroxytetrahydro-2H-pyran-2-yl)oxy)tetrahydro-2H-pyran-3-yl)oxy)-6-(hydroxymethyl)tetrahydro-2H-pyran-3,4,5-triol |
| 32 | sibiricoside A_qt | (4S,6aR,10R)-10-((R)-3-hydroxybutyl)-10-methoxy-6a-methyl-3,4,5,6,6a,6b,7,8,8a,8b,9,10,11a,12,12a,12b-hexadecahydro-1H-naphtho[2',1':4,5]indeno[2,1-b]furan-4-ol |
| 33 | sibiricoside B | (2S,3S,4S,5R,6R)-2-(((2S,3S,4S,5S,6R)-2-(((2R,3R,4R,5S,6R)-6-(((3'S,4S,5'R,6aS,8aS,9S,12aR,12bS)-3',12a-dihydroxy-5',6a,8a,9-tetramethyl-1,3,3',4,4',5,5',6,6a,6b,6',7,8,8a,8b,9,11a,12,12a,12b-icosahydrospiro[naphtho[2',1':4,5]indeno[2,1-b]furan-10,2'-pyran]-4-yl)oxy)-4,5-dihydroxy-2-(hydroxymethyl)tetrahydro-2H-pyran-3-yl)oxy)-5-hydroxy-6-(hydroxymethyl)-4-(((2R,3R,4R,5R)-3,4,5-trihydroxytetrahydro-2H-pyran-2-yl)oxy)tetrahydro-2H-pyran-3-yl)oxy)-6-(hydroxymethyl)tetrahydro-2H-pyran-3,4,5-triol |
| 34 | sibiricoside B_qt | (3'S,4S,5'R,6aR,6bR,8bS,9R,11aR,12aS)-5',6a,8a,9-tetramethyl-1,3',4,4',5,5',6,6a,6b,6',7,8,8a,8b,9,11a,12,12b-octadecahydrospiro[naphtho[2',1':4,5]indeno[2,1-b]furan-10,2'-pyran]-3',4,12a(3H)-triol |
| 35 | (+)-Syringaresinol-O-beta-D-glucoside | (2S,3R,4S,5S,6R)-2-(4-((1S,3aR,4S,6aR)-4-(4-hydroxy-3,5-dimethoxyphenyl)tetrahydro-1H,3H-furo[3,4-c]furan-1-yl)-2,6-dimethoxyphenoxy)-6-(hydroxymethyl)tetrahydro-2H-pyran-3,4,5-triol |
| 36 | vitexin xyloside | 5,7-dihydroxy-2-(4-hydroxyphenyl)-8-((2R,3R,4S,5S,6S)-3,4,5-trihydroxy-6-((((2S,3R,4R,5S,6R)-3,4,5,6-tetrahydroxytetrahydro-2H-pyran-2-yl)oxy)methyl)tetrahydro-2H-pyran-2-yl)-4H-chromen-4-one |
| 37 | (Z)-nonadec-6-enoic acid | (Z)-nonadec-6-enoic acid |
| 38 | zhonghualiaoine 1 | (1S,3R,4aS,6bR,9R,9aR,11bR)-1,3-dihydroxy-9-((R)-1-((1R,2R,3R)-2-isopropyl-3-methylcyclopropyl)propan-2-yl)-9a,11b-dimethyltetradecahydrocyclopenta[1,2]phenanthro[8a,9-b]oxiren-8(2H)-one |
| 39 | 2,3-Diaminobutyric Acid | (3S,4S)-3,4-diaminopentan-2-one |
| 40 | L-Î‘,Î“-Diaminobutyric Acid | (R)-2,4-diaminobutanoic acid |
| 41 | 3-Ethoxymethyl-5,6,7,8-Tetrahydro-8-Indolizinone | 3-(ethoxymethyl)-6,7-dihydroindolizin-8(5H)-one |
| 42 | Isomucronulatol | (R)-3-(2-hydroxy-3,4-dimethoxyphenyl)chroman-7-ol |
| 43 | Kinganone | 3-(butoxymethyl)-6,7-dihydroindolizin-8(5H)-one |

**Table S2** Core interaction targets identified through protein-protein interaction (PPI) analysis. This table lists 60 core targets with a degree ≥ 10 based on PPI network analysis, which may play key roles in the therapeutic effects of HJ for HUA.

| Number | Target | Number | Target |
| --- | --- | --- | --- |
| 1 | ALB | 31 | CDK6 |
| 2 | TNF | 32 | REN |
| 3 | CASP3 | 33 | CYP2D6 |
| 4 | BCL2 | 34 | CCND2 |
| 5 | PPARG | 35 | LDHA |
| 6 | PTGS2 | 36 | FASN |
| 7 | SIRT1 | 37 | CYP1A2 |
| 8 | PARP1 | 38 | CD38 |
| 9 | JAK2 | 39 | CYP2C9 |
| 10 | ABCG2 | 40 | TYMS |
| 11 | BCL2L1 | 41 | PDGFRA |
| 12 | ICAM1 | 42 | TOP2A |
| 13 | CXCL8 | 43 | CYP2C19 |
| 14 | MCL1 | 44 | SERPINE1 |
| 15 | MDM2 | 45 | NLRP3 |
| 16 | ABL1 | 46 | CCND3 |
| 17 | HDAC1 | 47 | APEX1 |
| 18 | CYP3A4 | 48 | LCK |
| 19 | MAPK1 | 49 | ALK |
| 20 | CDK4 | 50 | ADA |
| 21 | SYK | 51 | PNP |
| 22 | XDH | 52 | TLR9 |
| 23 | MAPK8 | 53 | ABCC1 |
| 24 | IGF1R | 54 | SMO |
| 25 | CYP1A1 | 55 | TOP1 |
| 26 | CCNA2 | 56 | SLC6A3 |
| 27 | CDK1 | 57 | LIPE |
| 28 | G6PD | 58 | PLAT |
| 29 | MAOA | 59 | DRD2 |
| 30 | HNF4A | 60 | PYGB |
